# Gegen Qinlian decoction enhances the effect of PD-1 blockade in colorectal cancer with microsatellite stability by remodelling the gut microbiota and the tumour microenvironment

**DOI:** 10.1038/s41419-019-1638-6

**Published:** 2019-05-28

**Authors:** Ji Lv, Yitao Jia, Jing Li, Wentao Kuai, Yang Li, Fang Guo, Xinjian Xu, Zhaolong Zhao, Jian Lv, Zhongxin Li

**Affiliations:** 1 Second Department of surgery, The Fourth Hospital of Hebei Medical University, 050035 Shijiazhuang, China; 2 Department of Surgery, The First Hospital of Qinhuangdao, 066000 Qinhuangdao, China; 3 Third Department of Oncology, Hebei General Hospital, 050051 Shijiazhuang, China; 4 Department of Traditional Chinese Medicine, The Fourth Hospital of Hebei Medical University, 050017 Shijiazhuang, China; 5 College of Combine Traditional Chinese and Western Medicine, Hebei Medical University, 050017 Shijiazhuang, China; 6 Department of Pharmacology, Hebei Medical University, 050017 Shijiazhuang, China

**Keywords:** Cancer immunotherapy, Colon cancer

## Abstract

Therapeutic antibodies targeting PD-1 have made major breakthroughs in cancer treatment. However, the majority of colorectal cancer (CRC) cases are microsatellite stable (MSS) and do not respond to anti-PD-1-based immunotherapy. Combination therapy will be an ideal strategy to overcome this limitation. Gegen Qinlian decoction (GQD), a classical traditional Chinese medicine (TCM) formula, has been clinically proven to be effective in the treatment of ulcerative colitis (UC) and type 2 diabetes mellitus. Here, a systemic pharmacological study revealed that GQD acts through multiple targets and pathways in the human body. Combination therapy with GQD and anti-mouse PD-1 potently inhibited the growth of CT26 tumours in a xenograft model. Gut microbiota analysis revealed that combination therapy with GQD and anti-mouse PD-1 significantly enriched for *s__Bacteroides_acidifaciens* and *s__uncultured_organism_g__norank_f__Bacteroidales_S24-7_group*. Based on metabolomic analyses, profoundly altered metabolites were identified in the combination therapy group. Two metabolic signalling pathways, namely, glycerophospholipid metabolism and sphingolipid metabolism, were explored. In particular, we found that combination therapy with GQD and anti-mouse PD-1 significantly increased the proportion of CD8+ T cells in peripheral blood and tumour tissues. Direct treatment with GQD and anti-mouse PD-1 increased the expression of IFN-γ, which is a critical factor in antitumour immunotherapy. In addition, combination therapy with GQD and anti-mouse PD-1 downregulated PD-1 and increased IL-2 levels, suggesting that the combination therapy could effectively restore T-cell functions by suppressing inhibitory checkpoints. The application of the Chinese medicinal formula GQD with PD-1 blockade-based immunotherapy can be a novel therapeutic strategy for CRC patients with MSS tumours.

## Introduction

Immunotherapy via inhibitory receptor targeting by specific antibodies has been a key breakthrough. It has emerged as the most promising approach for treatment and improving outcomes in a variety of malignancies. Blocking inhibitory receptors with specific antibodies may reverse antitumour responses^1^. In contrast to the proportion of patients with other tumour types, such as melanoma and lung cancer, the proportion of colorectal cancer (CRC) patients who benefit from immunotherapy is small, and only subgroups with deficient mismatch repair (dMMR) or microsatellite instability-high (MSI-H) CRC are amenable to checkpoint inhibition^2^. For the much larger subgroup of non-dMMR/MSI-H patients, combination regimens are urgently required and may be an ideal strategy to overcome this limitation^3^.

In traditional Chinese medicine (TCM), combination therapy has been advocated for thousands of years, and it is a unique ancient Chinese medical science for treating various diseases^4^. Gegen Qinlian decoction (GQD) is a well-known classical TCM for diarrhoea related to damp-heat syndrome for ~2000 years. GQD is also used to treat type 2 diabetes mellitus (2-DM) with good results^5,6^. Moreover, GQD has been clinically proven to be effective in the treatment of ulcerative colitis (UC)^6^. Wang et al.^7^ reported that GQD can suppress the expansion of human renal carcinoma by inhibiting MMP-2. In vivo and in vitro experiments have shown that several active components of GQD, such as baicalin, glabridin and berberine, can significantly alleviate inflammation and oxidative stress^8–11^.

It is well-known that long-standing UC can lead to the accumulation of high levels of pro-inflammatory cytokines within the colonic mucosa and thus lead to dysplastic lesions and cancer^12^, contributing substantially to the morbidity and mortality associated with this disease^13^. Among CRC patients, the majority of the population is MSS and does not respond to PD-1-based immunotherapy. A combination regimen will be an ideal strategy to overcome this limitation. Therefore, we hypothesised that GQD can exert antitumour effects against CRC and that it can be used in combination with immunotherapeutic agents for MSS patients. In the present study, the goal of a systemic pharmacological investigation was to uncover the targets and functions of GQD and anti-mouse PD-1 combination therapy. Then, we explored the effects of GQD and anti-mouse PD-1 combination therapy on xenograft tumours in mice induced using the mouse colorectal carcinoma cell line colorectal 26 (CT26), which is known to be MSS. Additionally, the effects of GQD and anti-mouse PD-1 combination therapy on mouse colorectal carcinoma were further assessed by evaluating the gut microbiota, metabolomic profiles, and the tumour microenvironment (TME). Our extensive study provides experimental evidence regarding combination therapy with GQD and anti-mouse PD-1 and highlights new treatment strategies for CRC.

## Methods

### Gegen Qinlian decoction (GQD) preparation

The herbal formula GQD is a combination of four medicinal herbs: *Radix Puerariae* (20 g)*, Scutellariae Radix* (12 g), *Coptidis Rhizoma* (12 g), and *liquorice* (8 g) at a rate of 5:3:3:2 (w/w/w/w). The four herbs that constitute GQD were purchased from Le-Ren-Tang (Shijiazhuang, China) and were identified by two experienced pharmacists. For extraction of GQD, the herbs were first soaked in 75% ethanol at eightfold volume (v/w) overnight and then extracted by decoction two times, 1.5 h for the first time and 1 h for the second time with sixfold volume of 75% ethanol to herbs (v/w). After filtration, the solution was evaporated under reduced pressure to obtain an extract, and, then the extract was evaporatedesiccated to powder at 60 °C and stored at 4 °C for further use.

### Component analysis of GQD powder with HPLC-MS/MS

Standard puerarin, berberine hydrochloride and baicalin were purchased from the National Institute for the Control of Pharmaceutical and Biological Products (Beijing, China). Standard daidzin, wogonoside and liquiritin were purchased from Weikeqi Biological Technology Co., Ltd. (Chengdu, China). Purified water used for the HPLC-MS system was obtained from the Hangzhou Wahaha Group Co., Ltd. (Hangzhou, China). LC/MS grade formic acid and ammonium acetate were obtained from Thermo Fisher Scientific Ltd. (St. Louis, MO, (USA). Acetonitrile and methanol used in the method were of HPLC grade and obtained from the Tedia Company (Cincinnati, Ohio, USA). Fifty milligrams of GQD powder was accurately weighed and extracted with 20 mL of 80% methanol in an ultrasonic bath for 20 min. The extraction solution was diluted five times and filtered by a 0.22 μm microporous membrane for HPLC-MS/MS analysis. The analysis was performed on a 3200 QTRAP^TM^ system with an electrospray ionisation (ESI) source operated in the positive ionisation mode with an Agilent 1200 HPLC system. All analytes were quantitated in the ion multiple reaction monitoring (MRM) mode. The precursor-to-product ion pairs, declustering potential (DP) and collision energy (CE) for each analyte are shown in Supplementary Table S1. Chromatographic separation was carried out on a Sonoma C18(2) (3 u 100 A 150 × 2.1 mm) column. The column temperature was maintained at room temperature, and the injection volume was 5 μL. The mobile phase consisted of acetonitrile (A) and water (B) (containing 0.1% formic acid and 5 mM ammonium acetate) with a flow rate of 0.3 mL/min. A gradient programme was used as follows: 0–6 min, 12% A, 6–9 min, 12% A-25% A, 9–13 min, 25% A, 13–14 min 25% A-27% A, 14–25 min, 27% A.

### Systemic pharmacological analysis of GQD

According to calculations and parameters by Wang^14^ and others^15,16^, the candidate compounds of GQD were screened out based on oral bioavailability (OB) >30% and drug-likeness (DL) >0.18. We then identified the potential targets for the candidate compounds in GQD using the systematic drug targeting approach developed by Yu et al.^17^. Moreover, known CRC-related targets were identified from three existing resources: (1) We searched for the keyword ‘colorectal cancer’ in the OMIM database (Online Mendelian Inheritance in Man; http://www.omim.org/) and obtained 47 targets^18^; (2) we searched for the keyword ‘colorectal cancer’ in Genetic Association Database (GAD; http://geneticassociationdb.nih.gov/) and obtained 111 known targets^19^; and (3) we searched for the keyword ‘colorectal cancer’ in the TTD database (Therapeutic Target Database; https://db.idrblab.org/ttd/)^20^ and obtained seven known targets. The detailed information of 150 non-redundant targets is given in Supplementary Table S2.

Subsequently, the compound-target network and disease-target network were constructed and visualised using Cytoscape (Version 3.5.0). Moreover, the protein-protein interaction (PPI) network was obtained using BioGRID (Biological General Repository for Interaction Datasets), and the PPI networks were further visualised using Cytoscape (available at http://www.cytoscape.org/)^21^. After we merged the PPI network for the targets of compounds and the PPI network for targets of disease, we extracted the hub network based on the topological property of each node in the interaction network, namely, degree centrality (DC), betweenness centrality (BC), closeness centrality (CC) and network centrality (NC).

### Functional annotation of key targets

The Database for Annotation, Visualisation and Integrated Discovery (DAVID) is the most common tool to analyse the functional enrichment of genes. To identify the functions of key targets, we used DAVID to perform KEGG pathway enrichment analyses. The *P*-value was calculated and further corrected using the Benjamini–Hochberg method, and an FDR <0.05 was selected as the cutoff criterion.

### Xenograft tumour transplantation model

The animal use protocol listed below has been reviewed and approved by the Laboratory Animal Ethical Committee Fourth Hospital Hebei Medical University. In the present study, we purchased a total of 96 BALB/c mice (male, ~20 g, aged 5 weeks). All the animals were fed adaptively for 1 week under specific pathogen-free conditions with food and water provided ad libitum, and then orally gavagedgavaged with 300 mg/kg (low-dose group), 1500 mg/kg (medium-dose group) or 7500 mg/kg GQD (high-dose group) once a day for 10 days. For the control group, 10 m/kg of vehicle (0.5% CMC-Na solution) was orally gavaged into micefor 10 days.

The mouse colorectal carcinoma cell line CT26 was cultured in RPMI 1640 medium (Gibco, Gaithers- burg, MD, USA) supplemented with 10% heat-inactivated foetal calf serum at 37 °C in a 5% CO_2_ incubator. CT26 cells (~2.5 × 10^6^ cells/mouse) were transplanted subcutaneously into the left axillary region of each mouse and allowed ~1 week to establish tumours. When the tumours reached a size of 50 mm^3^, the mice were intraperitoneally (i.p.) injected with 250 μg of anti-mouse PD-1 mAb (Bioxcell, Lebanon, NH, USA). The mice in the control group were administered the same volume of PBS. All mice were injected five times at 3-day intervals with anti-mouse PD-1 mAb or PBS, and tumour diameters were routinely measured using a caliper. Tumour volume was estimated as follows: length × width^2 ^× 0.5. Moreover, the tumour growth inhibition rate (TGI, %) was calculated as follows: TGI (%) = [1 – (tumour volume in the treated group)/(tumour volume in the control group)] × 100. GQD was administered without interruption throughout the animal experiment.

### Microbial analysis of mouse stool

Faeces of all mice were collected before and after anti-PD-1 immunotherapy for gut microbiota analyses. Briefly, (i) genomic DNA was extracted using a PowerSoil DNA Isolation Kit (MO BIO Laboratories, Carlsbad, CA); (ii) the 16S rDNA V4 region was amplified using the 515F and 806R primers; (iii) PCR product quantification, qualification and purification were performed; (iv) library preparation and sequencing were performed on the MiSeq platform (Illumina, Inc, San Diego, CA).

The 16S rRNA sequencing data were quality filtered using FLASH (Fast Length Adjustment of Short reads, Version 1.2.11). Operational taxonomic units (OTUs) were picked at a 97% sequence similarity cut-off, and the identified taxonomy was then aligned using Silva (Release128 http://www.arb-silva.de). Moreover, the RDP classifier (version 2.2) was used to classify OTUs at a given taxonomic rank.

### Untargeted analysis of mouse blood by ultra performance liquid chromatography-tandem mass spectrometer (UPLC-MS)

Whole blood was collected by removal of eyeball after anti-PD-1 immunotherapy. Plasma samples were obtained by centrifugation and stored at –80 °C. The plasma samples were precipitated as follows. Precipitated samples were injected onto a Waters HSS T3 column using a Waters ACQUITY™ UPLC system equipped with a Waters Xevo™ G2-XS Qtof for further analysis. For non-targeted metabolomic analyses, a gradient of 0.1% formic acid in water (A) and 0.1% formic acid in acetonitrile (B) was used. The optimal conditions for mass spectrometry were as follows: capillary voltage at 2.5 kV for the positive ion mode, cone voltage of 24 V, source temperature of 100 °C, desolvation gas flow of 800 L/h, and cone gas flow of 50 L/h. In the negative ion mode, the mass spectrometry parameters were as follows: capillary voltage of 2.5 kV, cone voltage of 25 V, source temperature of 100 °C, desolvation gas flow of 600 L/h, and cone gas flow of 10 L/h. The scan range was from 50 to 1500 m/z.

For effective separation of different lipid species, acetonitrile/H_2_O (60:40, v-v) mixed with 10 μM ammonium formate and 0.1% formic acid (A) and isopropanol/acetonitrile (90:10, v-v) mixed with 10 μM ammonium formate and 0.1% formic acid (B) were used. The optimal conditions for mass spectrometry were as follows: capillary voltage of 2.5 kV for the positive ion mode, cone voltage of 25 V, source temperature of 100 °C, desolvation gas flow of 600 L/h, and cone gas flow of 10 L/h. In the negative ion mode, the mass spectrometry parameters were as follows: capillary voltage of 2 kV, cone voltage of 40 V, source temperature of 100 °C, desolvation gas flow of 800 L/h, and cone gas flow of 50 L/h. The scan range was from 100 to 2000 m/z. Waters MassLynx v4.1 was used for all acquisition and analysis of data.

### Flow cytometry

Peripheral blood was collected from the orbital vein plexus with EDTA-Li micro-anticoagulant tubes. Blood was stained with FITC anti-mouse CD3-FITC, anti-mouse CD4-PE, and anti-mouse CD8-PE (BD Biosciences, San Jose, CA) antibodies. Acquisition was carried out on a Fortessa Flow Cytometer (BD Biosciences, San Jose, CA). Analysis was performed with FlowJo version 10 (Tree Star Inc., Ashland, OR).

### Immunohistochemistry and immunofluorescence on formalin-fixed paraffin-embedded (FFPE) samples

Xenograft tumours were harvested and embedded in paraffin blocks and cut into 4 µm thick tissue sections. The presence of tumour was confirmed on haematoxylin & eosin (H&E) staining. For immunohistochemical staining, paraffin sections on slides were dewaxed using xylene and rehydrated using alcohol at graded concentrations. Endogenous peroxidase activity was eliminated blocked using 3% H_2_O_2_ for 15 min. The slides were then blocked with 5% goat serum for 20 min at 37 °C, followed by primary antibody incubation overnight at 4 °C. The next day, each sample was incubated with horseradish peroxidase-labelled secondary antibody for 1 h at room temperature, followed by staining using a ready-to-use reagent DAB kit. After dehydrating and drying, the sections were mounted with neutral gum and observed under a microscope.

For immunofluorescence, xenograft tumours were embedded and cut as mentioned above. After antigen retrieval, the slides were incubated with primary antibody against CD8 overnight at 4 °C, followed by incubation with FITC-conjugated secondary antibody and staining with DAPI. Confocal fluorescence images were acquired with a laser scanning microscope (LSM 700; Zeiss, New York, NY) using a 20 × objective and processed with ZEN 2009 software (Zeiss, CA).

### Enzyme-linked immunosorbent assay (ELISA) on FFPE samples

Xenograft tumours were harvested, and protein was extracted from tumour tissues. Commercially available ELISA kits (MultiSciences (LIANKE) Biotech Co., Ltd, Hangzhou, China) were used for mouse protein expression detection of IL-17, IL-2, IL-6, TGF-β, IFN-γ, programmed death-1 (PD-1), T-cell surface glycoprotein CD8 alpha chain (CD8A), and T-cell surface glycoprotein CD4 (CD4) levels according to the manufacturer’s instructions.

### Statistical analysis

Statistical calculations were performed using SPSS software (SPSS Inc., Chicago, IL, USA). Comparisons between two groups were assessed using Student’s unpaired *t*-tests. Adjustments for multiple comparisons were performed by ANOVA with the Benjamini–Hochberg false discovery rate procedure. A level of *P* < 0.05 was selected as the point of minimal statistical significance in every comparison.

## Results

### High-performance liquid chromatography-mass spectrometry (HPLC-MS/MS) analysis of GQD powder

Six main active components (puerarin and daidzin from *Radix Puerariae*, berberine from *Rhizoma Coptidis*, baicalin and wogonoside from *Radix Scutellariae*, and liquiritin from *Radix Glycyrrhizae*) in GQD powder were quantified using corresponding calibration curves of chemical standards (Supplementary Fig. S1). The detailed information regarding calibration curves, linear ranges, and contents of analytes is listed in Supplementary Table S3.

### Potential antitumour mechanisms of GQD for CRC

**Fig. 1:**
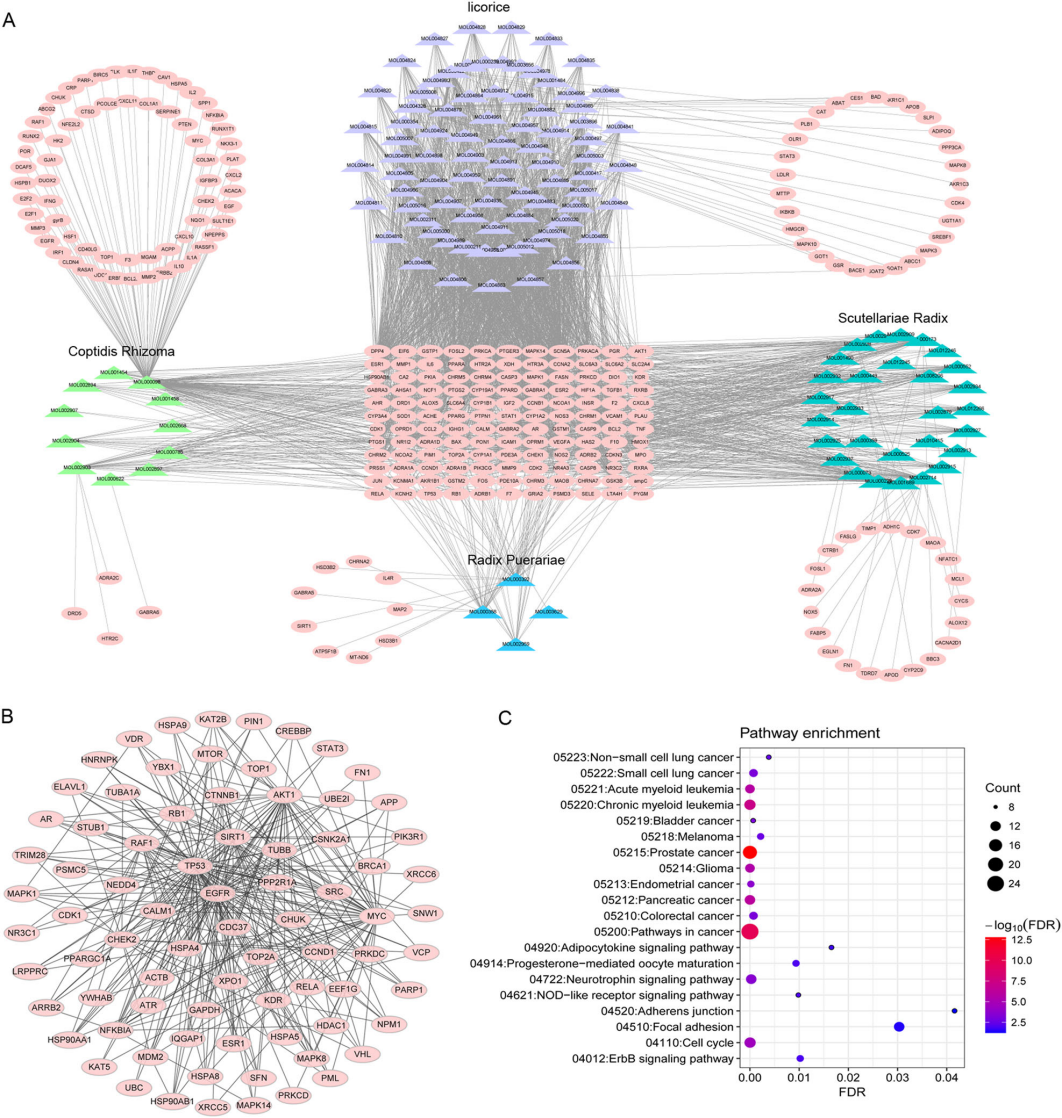
Potential antitumour mechanisms of GQD in colon cancer. **a** Construction of the GQD compound-putative target network. The compound-putative target network was constructed by linking candidate compounds from the four herbs *(Radix Puerariae, Scutellariae Radix, Coptidis Rhizoma* and *liquorice*), which are constituents of GQD, to their putative targets. The nodes representing candidate compounds are shown as polychromatic triangles, and the targets are indicated by pink squares. **b** PPI network of candidate GQD targets for colon carcinoma treatment extracted from the interactive PPI network of GQD putative targets and known colon carcinoma-related targets. **c** Pathway enrichment analysis of candidate targets for GQD in colon carcinoma

The chemical composition of all four herbs that constitute GQD was obtained from the Traditional Chinese Medicine System Pharmacology Database^22^. In total, GQD includes 476 chemicals, of which 18 are from *Radix Puerariae*, 143 are from *Scutellariae Radix*, 48 are from *Coptidis Rhizoma* and 280 are from *liquorice*. The information regarding the 476 chemicals is listed in Supplementary Table S4. By screening oral bioavailability (OB) and drug-likeness (DL), we obtained 138 potential active compounds from GQD, which were considered “candidate compounds”. The four herbs *Radix Puerariae*, *Scutellariae Radix, Coptidis Rhizoma* and *liquorice* contributed 4, 36, 14, and 90 candidate compounds, respectively. We next explored the therapeutic targets of the candidate compounds of GQD. In total, we obtained 260 potential therapeutic targets for 126 of 138 candidate compounds from GQD. Accordingly, a compound-putative target network for GQD was generated (Fig. 1a).

To explore the potential antitumour mechanisms of GQD for CRC, we further identified CRC-related targets from three databases, OMIM, GAD and TTD. In total, 150 CRC-related targets were obtained (Supplementary Table S2). Then, we constructed a PPI network for the putative targets of the compounds (6964 nodes and 18,776 edges) (Supplementary Fig. S2) and a PPI network for CRC-related targets (5572 nodes and 11,033 edges) (Supplementary Fig. S3). We intersected the two networks consisting of 3469 nodes and 5163 edges (Supplementary Fig. S4). Based on the median values for degree, betweenness centrality (BC) and closeness centrality (CC) values of 4, 0.000700195 and 0.3643827, respectively, we identified 79 significant targets for GQD in CRC (Fig. 1b).

The functional annotation indicated that the 79 significant targets of GQD may be involved in the pathology of a variety of tumours, such as CRC, lung cancer, prostate cancer, pancreatic cancer, endometrial cancer, bladder cancer, melanoma and glioma. Moreover, the cell cycle, focal adhesion and NOD-like receptor signalling pathway were enriched (Fig. 1c). Thus, we postulated that GQD exerts therapeutic effects on multiple targets and pathways of the human body through its complex active components.

### Antitumour efficacy of GQD and anti-mouse PD-1 combination therapy in vivo

**Fig. 2:**
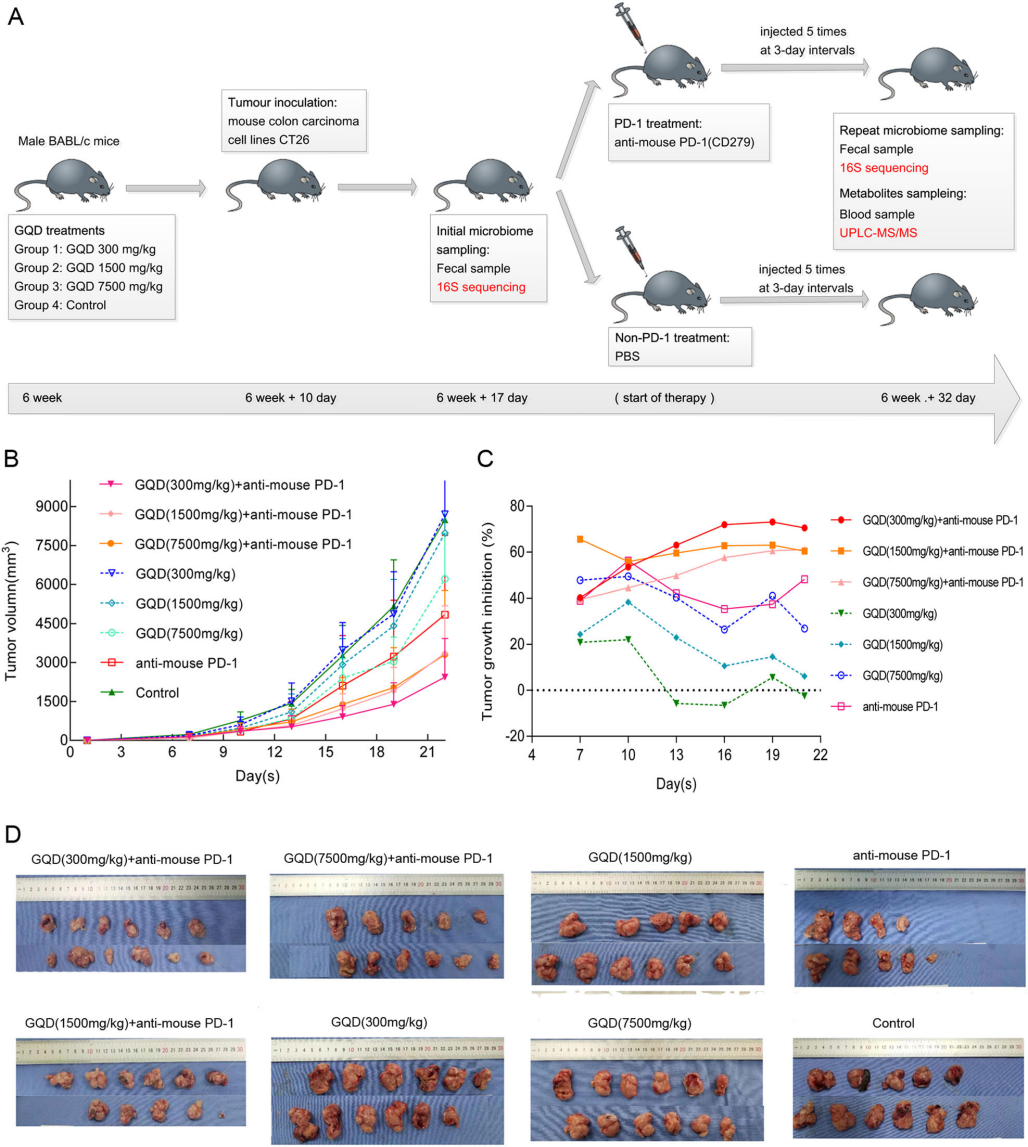
GQD and anti-mouse PD-1 combination therapy inhibits tumour growth of colon carcinoma in vivo. **a** Schema of sample collection and analysis. **b** Tumour volume changes in mice treated with GQD (300 mg/kg) + anti-mouse-PD-1, GQD (1500 mg/kg) + anti-mouse-PD-1, GQD (7500 mg/kg) + anti-mouse-PD-1, GQD (300 mg/kg), GQD (1500 mg/kg), GQD (7500 mg/kg), anti-mouse-PD-1 or normal saline (model) are shown. Data are presented as the mean ± SD. **c** Tumour growth inhibition rate (TGI) of mice. **d** Photographs of representative tumour blocks collected from mice from different treatment groups on 6 weeks + 32 days are shown

Combination regimens are urgently needed and may represent a great opportunity to overcome this challenge. Therefore, we hypothesised that GQD may be used in combination therapy with immunotherapeutic agents for CRC patients. In this study, we first investigated the in vivo effects of GQD and anti-mouse PD-1 combination therapy on the growth of colorectal carcinoma in CT26 tumour-bearing mice (Fig. 2a). Mice bearing subcutaneous CT26 xenografts in the 300 mg/kg GQD (low-dose), 1500 mg/kg GQD (medium-dose), 7500 mg/kg GQD (high-dose), PD-1, GQD (300 mg/kg) + PD-1, GQD (1500 mg/kg) + PD-1, and GQD (7500 mg/kg) + PD-1 groups treated for 32 days were compared with mice in the control group. The relative tumour volume on day 32 was 8.704 cm^3^ (low-dose group), 7.976 cm^3^ (medium-dose group), 6.216 cm^3^ (high-dose group), 4.847 cm^3^ (PD-1 group), 2.436 cm^3^ (GQD (300 mg/kg) + PD-1 group), 3.362 cm^3^ (GQD (1500 mg/kg) + PD-1 group), 3.305 cm^3^ (GQD (7500 mg/kg) + PD-1 group) and 8.496 cm^3^ (control group). However, compared with the control group, the low-dose GQD group showed no significant antitumour effect (*P* = 0.893). Compared with the control group, the PD-1 group displayed significantly inhibited growth of xenografted CT26 tumours (*P* = 0.002). In particular, tumour progression was dramatically inhibited by treatment with GQD and anti-mouse PD-1 combination therapy compared with the control (*P* = 0.000). GQD and anti-mouse PD-1 combination therapy significantly inhibited the growth of xenografted CT26 tumours compared with PD-1 alone (*P* = 0.034) (Fig. 2b). The tumour growth inhibition rate (TGI) on day 32 was –2.450% (low-dose group), 6.125% (medium-dose group), 26.836% (high-dose group), 48.216% (PD-1 group), 70.526% (GQD (300 mg/kg) + PD-1 group), 60.424% (GQD (1500 mg/kg/kg) + PD-1 group), and 60.097% (GQD (7500 mg/kg) + PD-1 group) (Fig. 2c). Based on the results of TGI, we found that the TGI of the high-dose GQD group was significant (Fig. 2c). However, the TGI of the low-dose GQD and anti-mouse PD-1 combination therapy group was the most significant (Fig. 2b–d). Altogether, these results showed that GQD enhances anti-PD-1 immunotherapy and can inhibit tumour growth of CRC in vivo.

### GQD modulates the gut microbiome composition

**Fig. 3:**
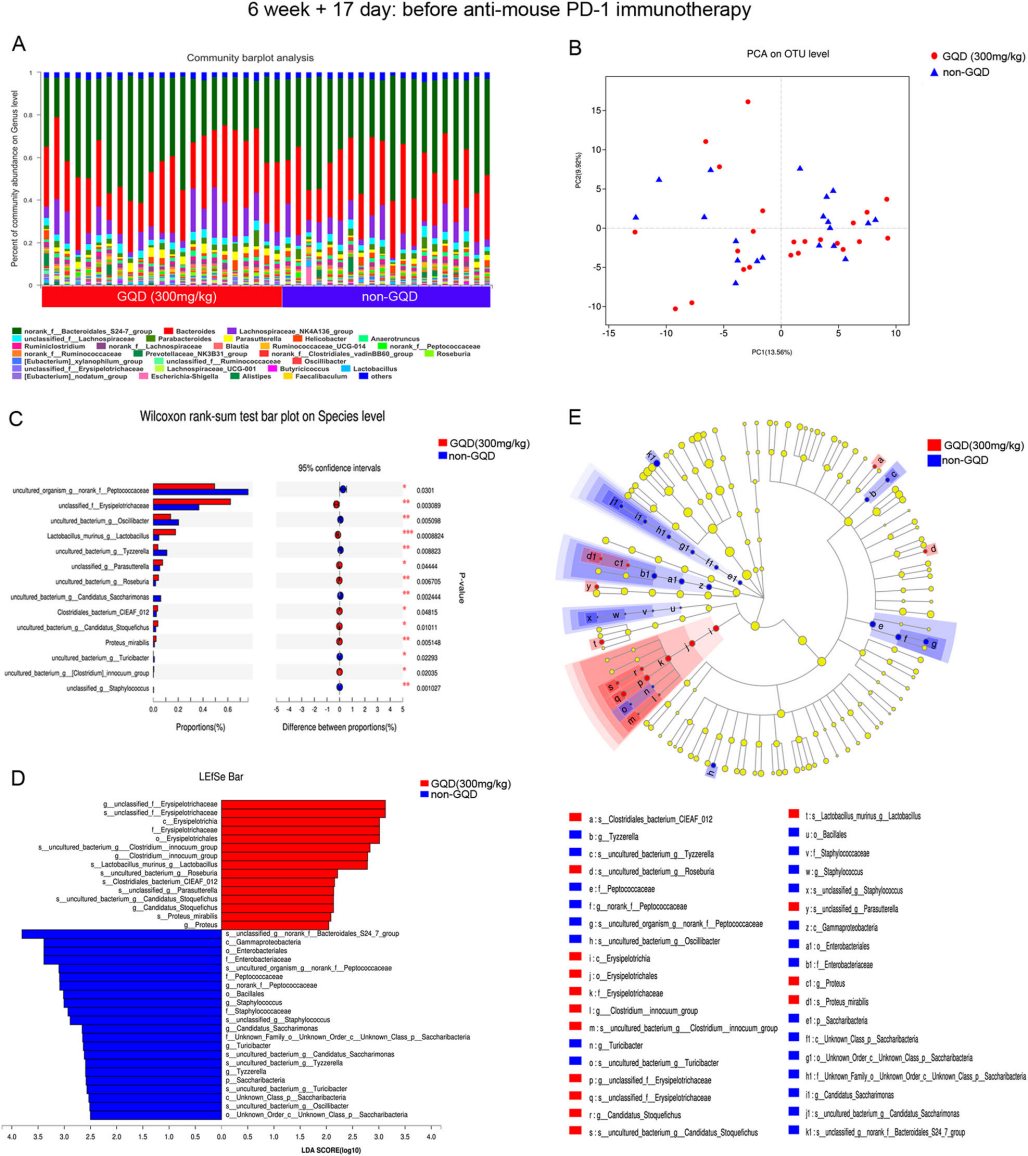
GQD modulates the gut microbiome composition. **a** Stacked bar plot of the phylogenetic composition of common bacterial taxa at the genus level in faecal samples at immunotherapy initiation. **b** Principal coordinate analysis (PCA) at the OTU level. **c** Bar plot of compositional differences at the species level in the gut microbiome of mice in the GQD (300 mg/kg) vs. the non-GQD group based on the Wilcoxon rank-sum test. **d** LDA scores computed for differentially abundant taxa in the faecal microbiomes from the GQD (300 mg/kg) and non-GQD groups. *P* = 0.05 for the Kruskal–Wallis test; LDA score > 2. **e** Taxonomic cladogram from LEfSe showing differences in faecal taxa. Dot size is proportional to the abundance of the taxon

After 17 days of administration of GQD, we prospectively collected gut (faecal) microbiome samples from CT26 tumour-bearing mice starting before treatment with anti-mouse PD-1 mAb therapy (*n* = 43), including samples from the low-dose GQD (300 mg/kg) (*n* = 23) and non-GQD (*n* = 20) groups. We first assessed the composition of the gut microbiome, noting relative diversity of two communities between the low-dose GQD group and the non-GQD group (Fig. 3a). Principal coordinate analysis (PCA) demonstrated that the microbial community structure was not clearly separated between the low-dose GQD group and the non-GQD group (Fig. 3b). Moreover, no significant differences were observed in the alpha diversity of the gut microbiome between the low-dose GQD group and the non-GQD group using several indices, such as Ace, Chao and Shannon (Supplementary Fig. S5). We then sought to determine if differences existed in the gut microbiomes between the low-dose GQD group and the non-GQD group. Interestingly, based on the Wilcoxon rank-sum test, when we compared the phylogenetic composition of common bacterial taxa at the species level, we found that *s__unclassified_f__Erysipelotrichaceae, s__Lactobacillus_murinus_g__Lactobacillus* and *s__unclassified_g__Parasutterella* were enriched in the low-dose GQD group and *s__uncultured_organism_g__norank_f__Peptococcaceae, s__uncultured_bacterium_g__Oscillibacter* and *s__uncultured_bacterium_g__Tyzzerella* were enriched in the non-GQD group (Fig. 3c). To further explore these findings, we performed high-dimensional class comparisons via linear discriminant analysis of effect size (LEfSe), which again demonstrated differentially abundant bacteria in the faecal microbiome between the low-dose GQD group and the non-GQD group, with *Erysipelotrichaceae* enriched in the low-dose GQD group and *Peptococcaceae* and *Bacteroidales_S24_7_group* enriched in the non-GQD group (Fig. 3d, e).

### GQD continuously regulates the gut microbiome and enhances the antitumour activity of anti-PD-1 antibody

**Fig. 4:**
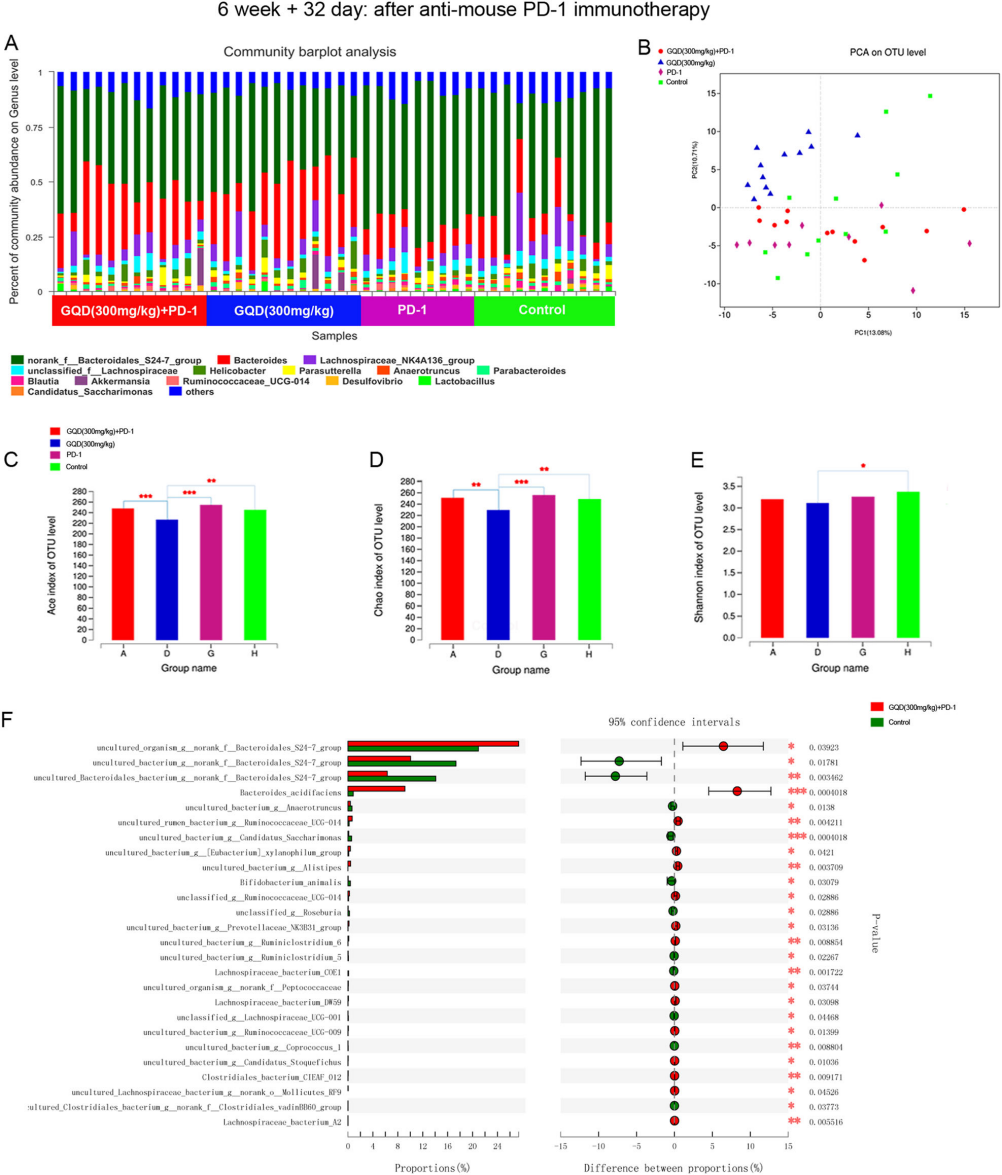
GQD continuously regulates the gut microbiome and enhances the antitumour activity of anti-PD-1 antibody. **a** Stacked bar plot of the phylogenetic composition of common bacterial taxa at the genus level in faecal samples after immunotherapy. **b** Principal coordinate analysis (PCA) at the OTU level. **c**, **d**, **e** Alpha diversity of the gut microbiome in the four groups using Ace, Chao and Shannon indices. **f** Bar plot of compositional differences at the species level in the gut microbiome of mice in the combination therapy group vs. the control group by the Wilcoxon rank-sum test

It is well-known that compositional differences in the microbiome may also influence cancer development and response to therapy. Therefore, we sought to determine if differences existed in the gut microbiomes between the GQD and non-GQD groups in response to anti-PD-1 therapy. To test this, we also collected gut microbiome samples from CT26 tumour-bearing mice (*n* = 44) at the end of immunotherapy (6 weeks + 32 days), including samples from the GQD (300 mg/kg) + PD-1 group (*n* = 12), GQD (300 mg/kg) group (*n* = 12), PD-1 group (*n* = 9) and control group (*n* = 11). By visualising the landscape of the gut microbiome in all available samples, we found that communities were relatively diverse in the four groups, with a high abundance of *g__Bacteroides* in the GQD and GQD + PD-1 combination therapy groups and *g__norank_f__Bacteroidales_S24-7_group* in the PD-1 and control groups (Fig. 4a). Importantly, PCA results also demonstrated a notable clustering effect in the gut microbiome of these four groups (Fig. 4b). We also found that the alpha diversity of the gut microbiome was significantly lower in the low-dose GQD group than in the other three groups based on Ace, Chao and Shannon indices (Fig. 4c–e). Pairwise comparisons were then performed for bacterial taxa at the genus level. The results indicated that *g__Bacteroides* was significantly enriched in the low-dose GQD and low-dose GQD and anti-mouse PD-1 combination therapy groups, *g__Anaeroplasma* was significantly enriched in the anti-mouse PD-1 group, and *g__norank_f__Bacteroidales_S24-7_group* was significantly enriched in the control group (Supplementary Fig. S6). At the species level, we observed that the combination therapy group was enriched in *s__Bacteroides_acidifaciens* and *s__uncultured_organism_g__norank_f__Bacteroidales_S24-7_group*, whereas the control group was enriched in *s__uncultured_bacterium_g__norank_f__Bacteroidales_S24-7_group* and *s__uncultured_Bacteroidales_bacterium_g__norank_f__Bacteroidales_S24-7_group* (Fig. 4f). Further high-dimensional class comparisons via LEfSe again demonstrated differentially abundant bacteria in the faecal microbiome (Supplementary Fig. S7). Importantly, the gut microbiome was shown to change continuously over time in a limited number of longitudinal samples tested (Figs. 3 and 4).

### GQD and anti-mouse PD-1 combination therapy induces specific changes in plasma lipids and metabolome

**Fig. 5:**
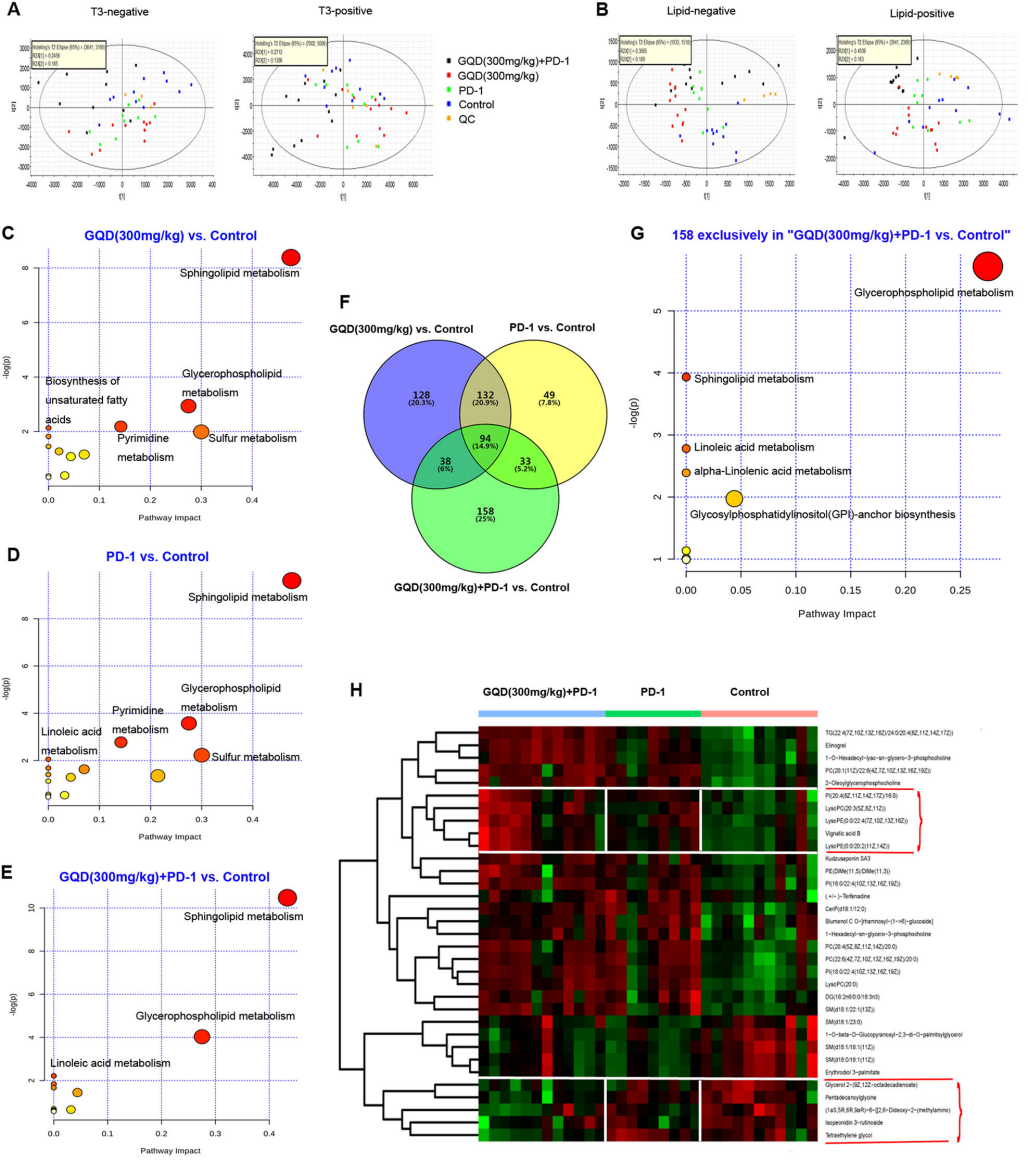
GQD and anti-mouse PD-1 combination therapy induces specific changes in plasma lipids and metabolome. **a** PCA plots of the plasma metabolome from all samples in the positive and B negative ion modes. **b** PCA plots of the plasma lipidome from all samples. **c**, **d**, **e** Pathway enrichment of differential metabolites in the GQD (300 mg/kg) + PD-1 group, GQD (300 mg/kg), or PD-1 group compared with the control group. **f** Venn diagram of differential metabolites in the four groups. **g** Pathway enrichment of 158 specific metabolites in the GQD (300 mg/kg) + PD-1 vs. control group. **h** Heatmap showing normalised relative abundances of metabolites, with annotation, that were significantly changed in the GQD (300 mg/kg) + PD-1 group vs. the control group and the PD-1 group vs. the control group but not in the GQD (300 mg/kg) group vs. the control group

We then performed plasma metabolite profiling on the same mice (*n* = 44) by UPLC-MS at the end of immunotherapy (6 weeks + 32 days), including samples from the GQD (300 mg/kg) + PD-1 group (*n* = 12), the GQD (300 mg/kg) group (*n* = 12), the PD-1 group (*n* = 9) and the control group (*n* = 11). PCA showed that QC samples clustered tightly in the score plots, indicating good data quality and reproducibility of the analytical methods (Fig. 5a, b). Moreover, PCA indicated significant differences in the distribution of plasma metabolites in the four groups (Fig. 5a, b). Metabolites with VIP values >1.0 and *P*-values < 0.05 were considered to be significantly changed. In total, 391, 308 and 322 metabolites were significantly changed in the GQD vs. control, PD-1 vs. control and GQD + PD-1 vs. control groups, respectively. Heatmaps also indicated that the plasma metabolites in the GQD, PD-1 and GQD + PD-1 groups were well separated from those in the control group (Supplementary Fig. S8).

Further pathway enrichment was performed to uncover the function of plasma metabolites. Figure 5c shows the enriched pathways for plasma metabolites comparing the GQD (300 mg/kg) group vs. the control group, indicating sphingolipid metabolism as the significant pathway. Figure 5d shows the enriched pathways for plasma metabolites comparing the PD-1 group vs. the control group, indicating sphingolipid metabolism and glycerophospholipid metabolism as the significant pathways. Figure 5e reveals the enriched pathways for plasma metabolite comparing the GQD (300 mg/kg) + PD-1 group vs. the control group, indicating sphingolipid metabolism and glycerophospholipid metabolism as the significant pathways. There were 158 specific metabolites in the GQD (300 mg/kg) + PD-1 group vs. the control group (Fig. 5f), which mainly affected the metabolic pathways of glycerophospholipid metabolism and sphingolipid metabolism (Fig. 5g). Next, we sought to explore how specific metabolites impact the response to anti-PD-1 therapy. Supervised hierarchical clustering was then performed using the 33 metabolites, which were common in the GQD (300 mg/kg) + PD-1 group vs. the control group and the PD-1 group vs. the control group but absent in the GQD (300 mg/kg) group vs. the control group (Fig. 5g). We focused on a group of metabolites with higher abundance in the GQD (300 mg/kg) + PD-1 group, including LysoPC(20:3(5Z,8Z,11Z)), vignatic acid B, LysoPE(0:0/22:4(7Z,10Z,13Z,16Z)), LysoPE(0:0/20:2(11Z,14Z)) and PI(20:4(8Z,11Z,14Z,17Z)/16:0). Moreover, we focused on another group of metabolites with lower abundance in the GQD (300 mg/kg) + PD-1 group, including (1aS,5R,6R,9aR)-6-{[2,6-dideoxy-2-(methylamino), isopeonidin 3-rutinoside, glycerol 2-(9Z,12Z-octadecadienoate), tetraethylene glycol and pentadecanoylglycine (Fig. 5g).

### GQD and anti-mouse PD-1 combination therapy enhances antitumour immunity

**Fig. 6:**
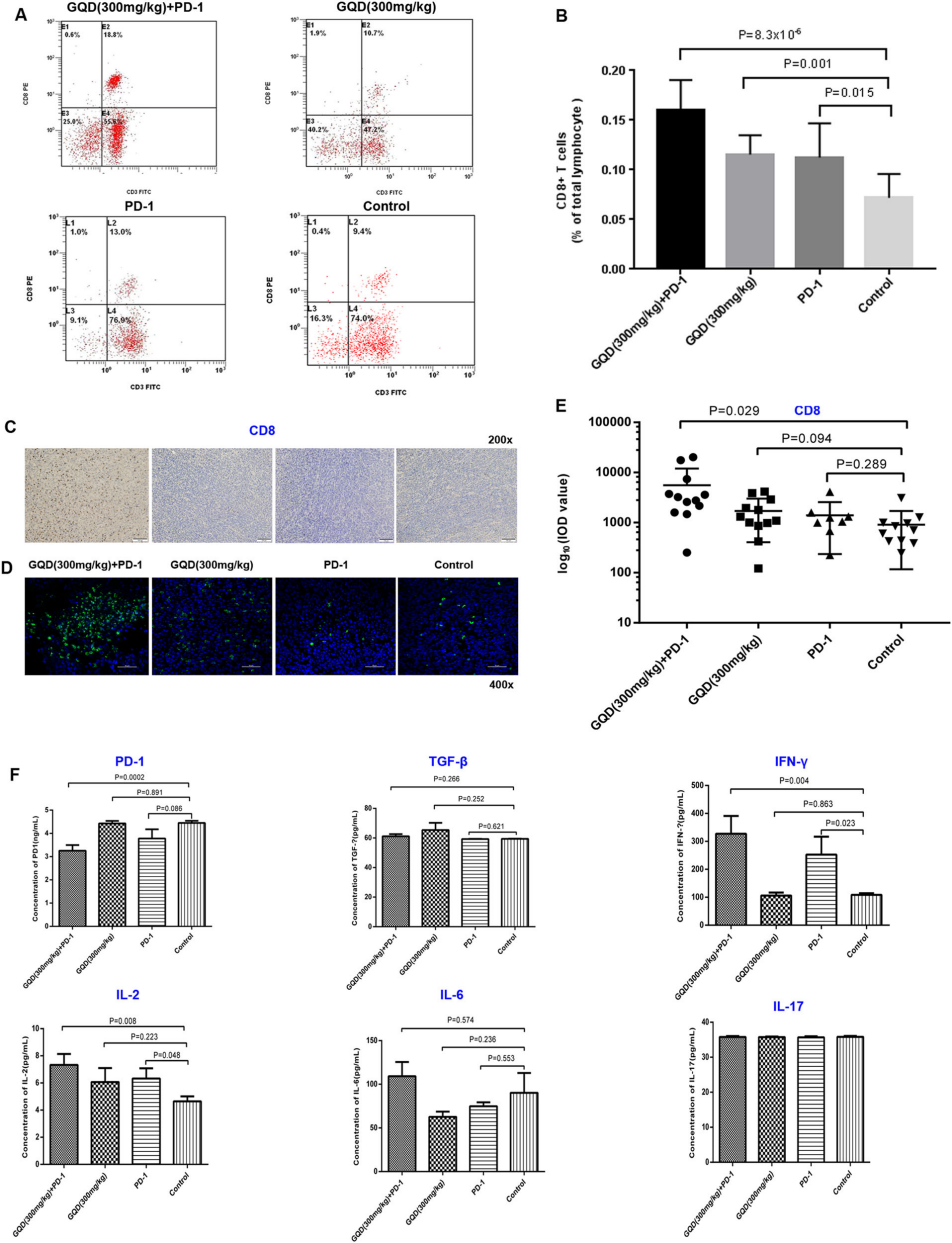
GQD and anti-mouse PD-1 combination therapy enhances systemic and antitumour immunity. **a** The proportion of CD8+ T-cell subpopulations among peripheral blood lymphocytes. **b** Bar graph of CD8+ T-cell subpopulations among peripheral blood lymphocytes. **c** Representative immunohistochemistry images and **d** immunofluorescence images of FFPE tumour samples. **e** Cluster plots of CD8+ T cells in tumour samples. **f** Bar graph of PD-1, TGF-β, IFN-γ, IL-2, IL-6, and IL-17 levels based on ELISA in tumour samples

Thirty-four tumour-bearing mice (four groups) were used, and peripheral blood lymphocyte cells were stained. Systemic immune responses were analysed via flow cytometry. The CD8+T-cell proportion among total peripheral blood lymphocyte cells was most significantly higher in the GQD and anti-mouse PD-1 combination therapy group than in the control group (Fig. 6a, b). Moreover, the CD8+T-cell proportion was significantly higher in the combination therapy group than in the PD-1 monotherapy group (*P* = 0.007). Additionally, the CD4+T cell proportion in the GQD and anti-mouse PD-1 combination therapy group tended to be lower than that in the control group (*P* > 0.05, data not shown).

There is clear evidence in preclinical models that changes in the tumour microenvironment are closely related to therapeutic responses to anti-PD-1 therapy. Thus, we next examined the tumour-associated immune infiltrates in tumour tissues after combination treatment with GQD and anti-mouse PD-1 via immunohistochemistry and immunofluorescence and observed a higher density of CD8+T cells in the GQD and anti-mouse PD-1 combination therapy group than in the control group (*P* = 0.029), consistent with the above results (Fig. 6c–e). The ELISA results revealed that mice after combination treatment with GQD and anti-mouse PD-1 had lower levels of PD-1 (*P* = 0.0002) and CD4 (*P* = 0.001). Meanwhile, these mice had higher levels of IL-2 (*P* = 0.008) and IFN-γ (*P* = 0.004). No significant changes were observed for TGF-β, CD8, IL-6 or IL-17 (Fig. 6f). These results suggested that combination therapy could effectively enhance the effect of PD-1 blockade in CRC with MSS tumours.

## Discussion

Systemic pharmacology is a method for understanding the dysregulation of signal transduction in diseases and for characterising the mode of action of drugs^23^. In the present study, we employed a comprehensive approach to clarify the synergistic effects and mechanisms of multi-component, multi-target agents in GQD, and this approach included combined prediction of active compounds and identification of multiple drug targets by network analysis. This analysis highlighted that GQD has 79 significant targets in CRC and inhibits CRC by regulating the cell cycle, focal adhesion and NOD-like receptor signalling pathway, which are induced by CRC-related oncogenes and dysregulated suppressor genes. Based on KEGG pathway enrichment, we also found that the T-cell receptor signalling pathway and Toll-like receptor signalling pathway were the top 30 significant pathways (results not shown). In non-small cell lung cancer, EGF receptor suppresses antitumour immunity by activating the PD-1 pathway to inhibit T-cell functions and increase pro-inflammatory cytokine levels^24^. Toll-like receptor signalling is involved in activating innate and adaptive immune responses and plays a critical role in inflammation-induced diseases, such as CRC^25^. Therefore, we speculated that targets of GQD were related to the antitumour immune response.

Nivolumab and pembrolizumab, two types of PD-1 inhibitors, were approved for the treatment of dMMR or MSI-H CRC in the United States in 2017, but this cancer type only accounts for a small subgroup of patients with CRC^3^. In addition to dMMR/MSI-H tumours, several approaches have been conducted to detect another 4 consensus molecular subtypes (CMS 1-4), which may also be susceptible to immunotherapy. However, there is no clinical relevance yet^26–29^. Therefore, improving the antigen-recognition efficiency of MSS-type CRC is a key issue for improving responses to checkpoint blockade immunotherapies. In the present study, we investigated the anticancer activity of GQD combined with anti-mouse PD-1 in a CT26 xenograft tumour transplantation model. The in vivo results indicated that the antitumour effect of GQD and anti-mouse PD-1 combination therapy was synergistically greater than that of monotherapy with either GQD or anti-mouse PD-1. These results suggest that GQD enhances anti-PD-1 immunotherapy for MSS-type CRC patients. Interestingly, the greatest antitumour activity of the combination therapy occurred in the low-dose GQD group rather than in the medium-dose or high-dose GQD group. The complex immune mechanisms involved need to be further explored.

CRC is a multifactorial disease associated with a variety of lifestyle aspects. Preclinical models have revealed that differential composition of the gut microbiome may influence therapeutic responses to anti-PD-1 therapy at the level of the tumour microenvironment^30^. Thus, we next examined gut microbiota modulation by GQD and anti-mouse PD-1 combination therapy. Redundancy analysis showed that the putative beneficial bacteria *g__Bacteroides* and the “double-response controller” of *g__norank_f__Bacteroidales_S24-7_group* were affected by combination treatment with GQD and anti-mouse PD-1. *Bacteroides* have been considered a potential marker for the early detection of CRC^31^. Herein, *Bacteroides acidifaciens* was significantly enriched in the GQD and anti-mouse PD-1 combination therapy group. *Bacteroides acidifaciens* is one of the predominant bacteria responsible for promoting IgA production in the large intestine^32^. In our study, we also proposed that *Bacteroides acidifaciens* is a “beneficial bacteria”, which may modulate metabolites and enhance host immunity. Some members of the Bacteroidales_S24-7_group have been shown to be IgA coated, suggesting that they may be targeted by the innate immune system^33,34^. The *s__uncultured_organism_g__norank_f__Bacteroidales_S24-7_group* was significantly enriched in the GQD and anti-mouse PD-1 combination therapy group, indicating that this bacteria may be necessary for antitumour immunotherapy. However, the other two bacteria types, s__uncultured_Bacteroidales_bacterium_g__norank_f__Bacteroidales_S24-7_group and s__uncultured_bacterium_g__norank_f__Bacteroidales_S24-7_group, were enriched in the control group, suggesting that they are probably “unfavourable”. Interestingly, a study in mice reported that the Bacteroidales_S24-7_group is a major bacterial group that might release bacterial extracellular DNA (eDNA), which can decrease pro-inflammatory activity and exert immunomodulatory functions in the mouse small intestine^35^. Our results further verified the dual function of the Bacteroidales_S24-7_group in its anticancer efficacy.

Metabolomics focuses on the investigation of global metabolites present in a biological specimen, which are considered representative of the phenotype^36^. In this study, the pathways of glycerophospholipid and sphingolipid metabolism were significantly changed in the GQD (300 mg/kg) + PD-1 group vs. the control group. It has been reported that glycerophospholipids and sphingolipids could be biomarkers for monitoring patients with CRC^37,38^. Hence, we speculated that GQD enhances the effect of PD-1 blockade in CRC by regulating the pathways of glycerophospholipid and sphingolipid metabolism. We found that LysoPC(20:3(5Z,8Z,11Z)), vignatic acid B, LysoPE(0:0/22:4(7Z,10Z,13Z,16Z)), LysoPE(0:0/20:2(11Z,14Z)) and PI(20:4(8Z,11Z,14Z,17Z)/16:0) were more abundant while another five metabolites ((1aS,5R,6R,9aR)-6-{[2,6-Dideoxy-2-(methylamino), isopeonidin 3-rutinoside, glycerol 2-(9Z,12Z-octadecadienoate), tetraethylene glycol and pentadecanoylglycine) were less abundant in the GQD+PD-1 group vs. the control group. Our results further supported that these metabolites could be a biomarker in monitoring patients with CRC.

The majority of CRC is not responsive to immunotherapy^39,40^. We found that combination treatment with GQD and anti-mouse PD-1 enhanced antitumour activity by promoting the infiltration of CD8+ T cells in tumour tissues, consistent with prior reports^41–43^. IFN-γ is a pleiotropic cytokine that is involved in all three phases of tumour immunoediting, such as elimination, equilibrium or dormancy, and escape, and it has a good prospect in overcoming tumour escape^44^. Mice receiving GQD and anti-mouse PD-1 combination therapy also had higher levels of IFN-γ in tumour tissues, suggesting improved host immune responses. We also observed that mice receiving GQD and anti-mouse PD-1 combination therapy had lower levels of PD-1 and higher levels of IL-2 in tumour tissues. Low PD-1 and high IL-2 levels are important indicators of the recovery function of anergic T cells^1^. In this study, combination therapy with GQD and anti-mouse PD-1 downregulated PD-1 and increased IL-2 expression, suggesting that combination therapy could effectively restore the T cell functions by suppressing the inhibitory checkpoints.

Our preclinical findings indicated that combination therapy with GQD and anti-mouse PD-1 mAb displays significant antitumour effects in vivo, indicating it a promising treatment option for a range of CRC subtypes. We proposed that combining GQD and anti-PD-1 drugs is a potential strategy in the field of immunotherapeutic drugs and TCM combination therapeutics, although further detailed laboratory investigations are needed to fully validate this promising combination.

## Supplementary information

Chromatograms of mixed standard solution (A) and sample solution (B). 1: puerarin; 2: daidzin; 3: liquiritin; 4: baicalin; 5: berberine; and 6: wogonoside

PPI network of GQD putative targets

PPI network of known colon cancer-related targets

The interactive PPI network of GQD putative targets and known colon cancer-related targets

Alpha diversity of the faecal microbiome before anti-mouse PD-1 immunotherapy

(A, B, C) Bar plot of compositional differences at the genus level in the gut microbiome of mice from each group. (D, E, F) LDA scores computed for differentially- abundant taxa in the faecal mic

Taxonomic cladogram from LEfSe showing differences in faecal taxa. Dot size is proportional to the abundance of the taxon

Unsupervised heatmaps of metabolites significantly changed in the GQD vs. control, PD-1 vs. control and GQD+ PD-1 vs. control groups

Monitoring of precursor-to-product ion pairs, declustering potential (DP) and collision energy (CE) of analytes

Known colon cancer-related targets

Linear regression data and contents of analytes

Compositive compounds of each compound in GQD

Supplementary figure legends
