# Supplementary figures and images for: Gegen Qinlian decoction enhances the effect of PD-1 blockade in colorectal cancer with microsatellite stability by remodelling the gut microbiota and the tumour microenvironment
Source: Cell Death Dis. 2019 May 28;10(6):415. doi: 10.1038/s41419-019-1638-6 (PMC6538740; doi:10.1038/s41419-019-1638-6)

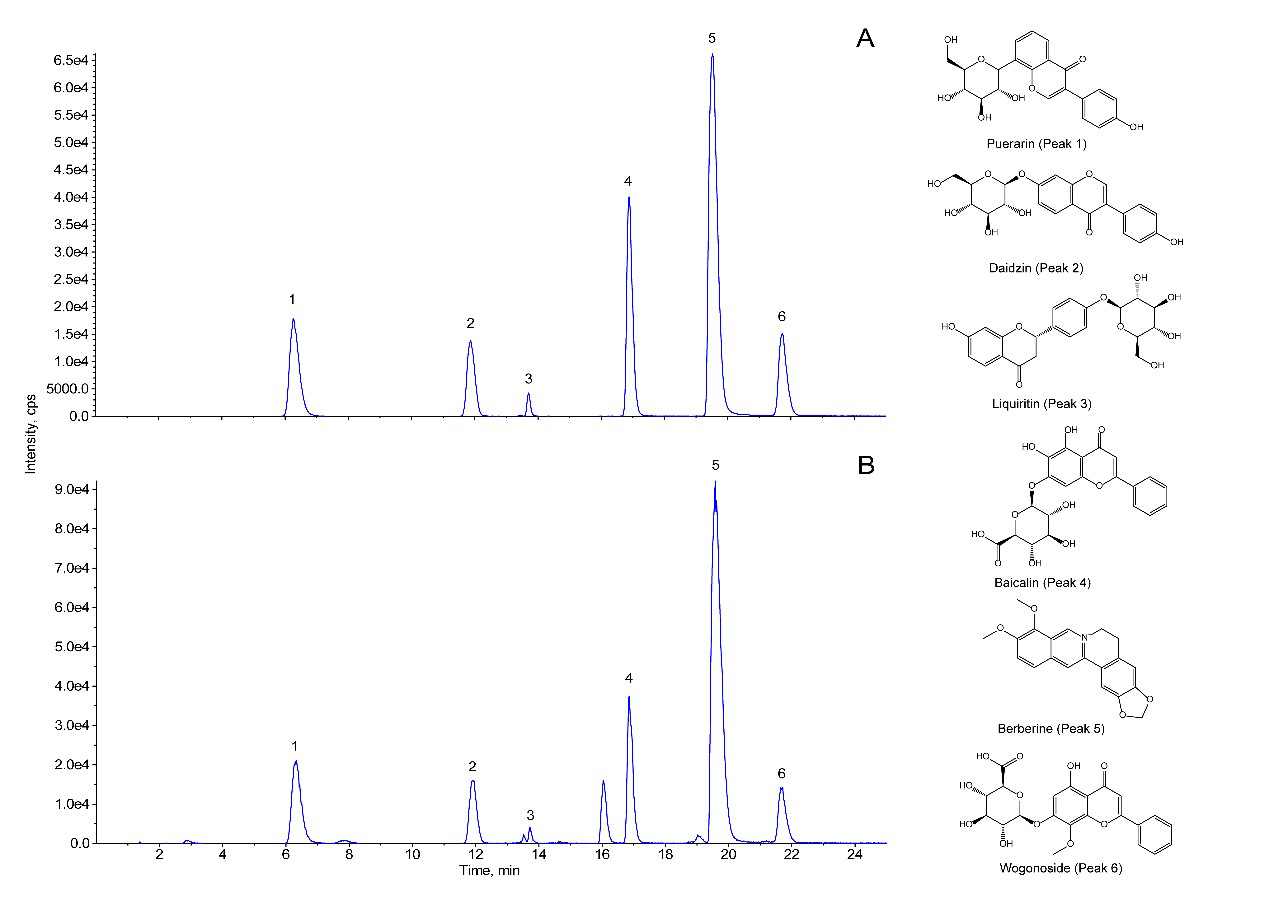

Supplement: Supplementary file 1 — Chromatograms of mixed standard solution (A) and sample solution (B). 1: puerarin; 2: daidzin; 3: liquiritin; 4: baicalin; 5: berberine; and 6: wogonoside [file 41419_2019_1638_MOESM1_ESM.tif]

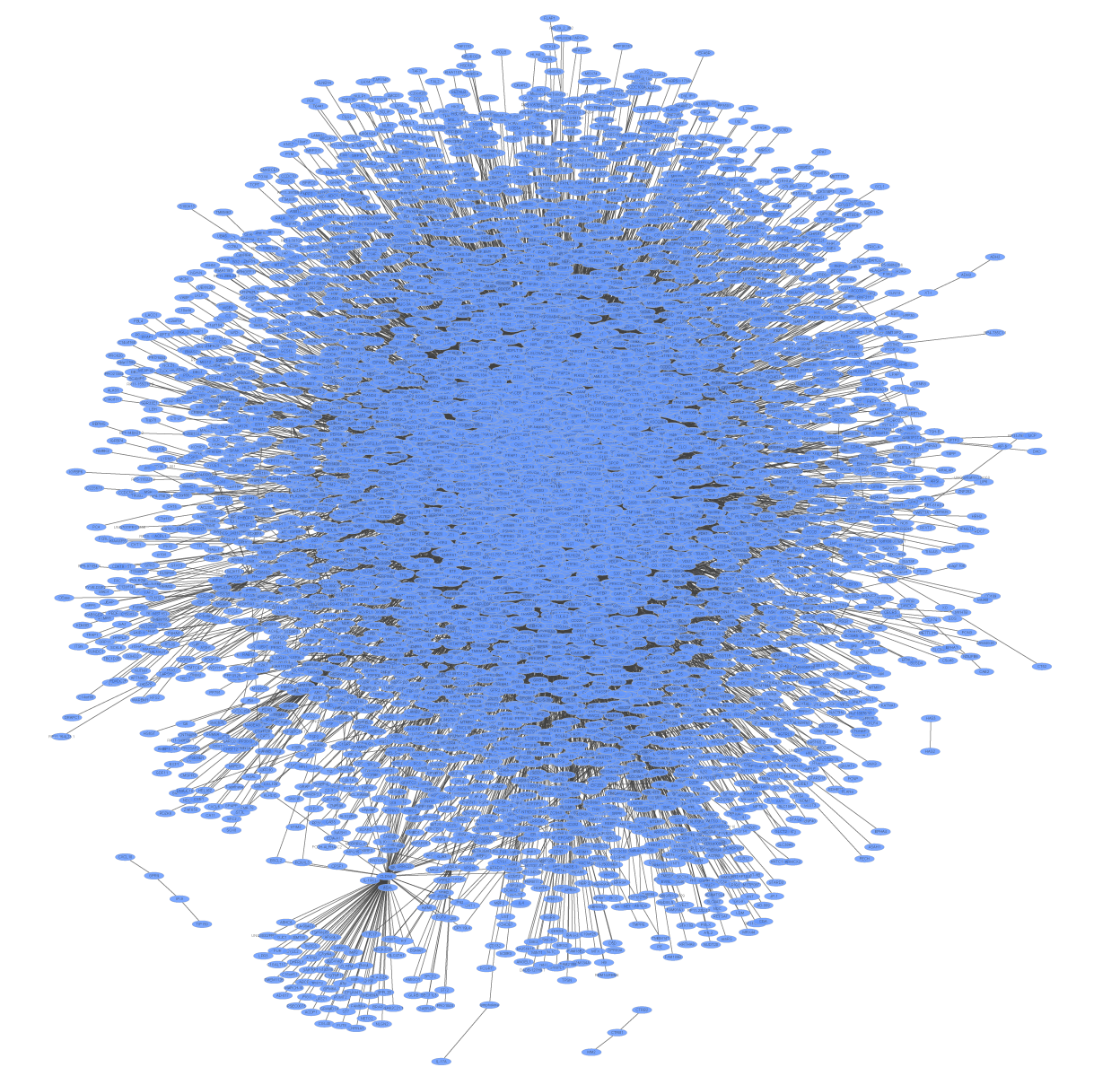

Supplement: Supplementary file 2 — PPI network of GQD putative targets [file 41419_2019_1638_MOESM2_ESM.tif]

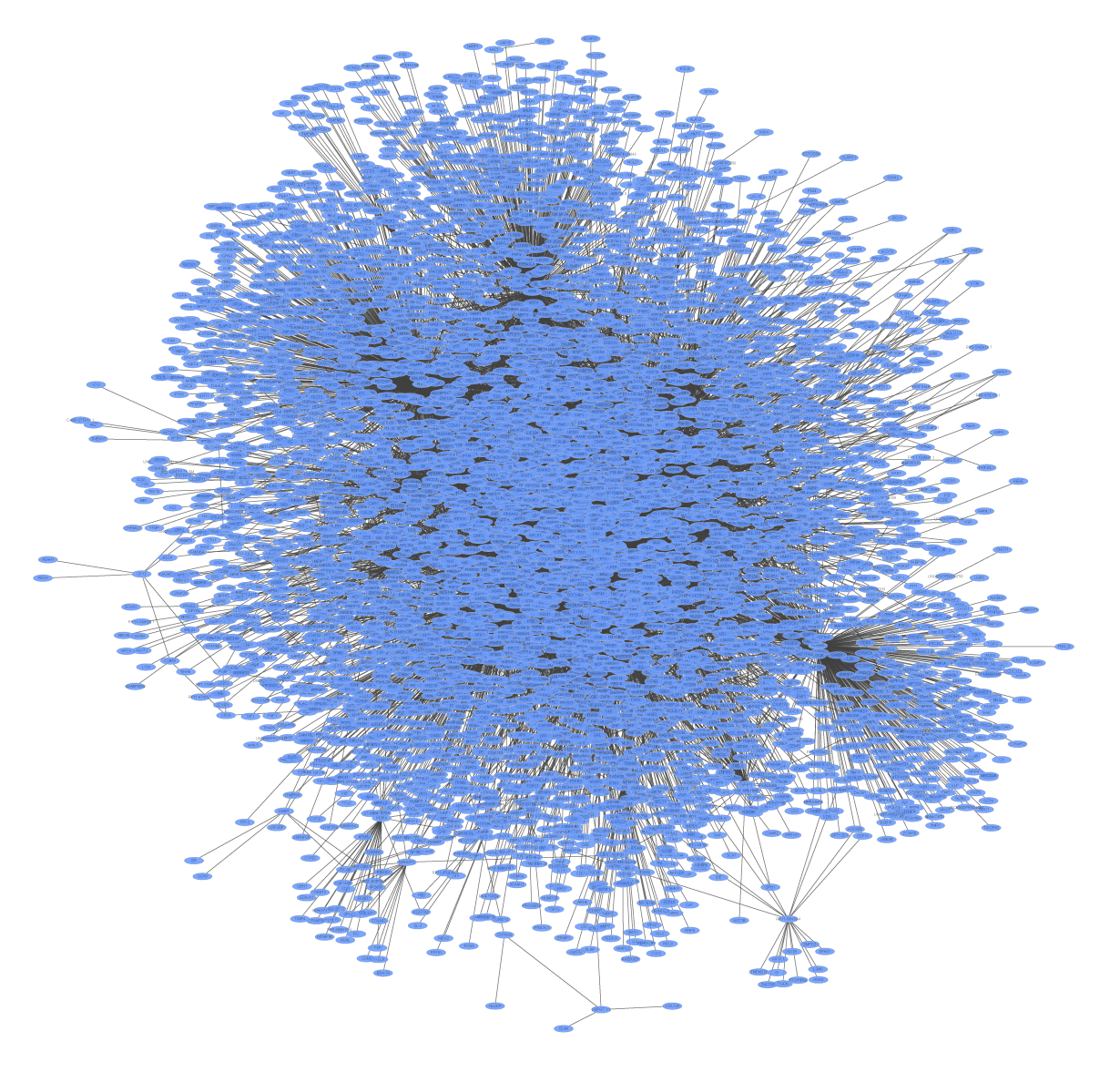

Supplement: Supplementary file 3 — PPI network of known colon cancer-related targets [file 41419_2019_1638_MOESM3_ESM.tif]

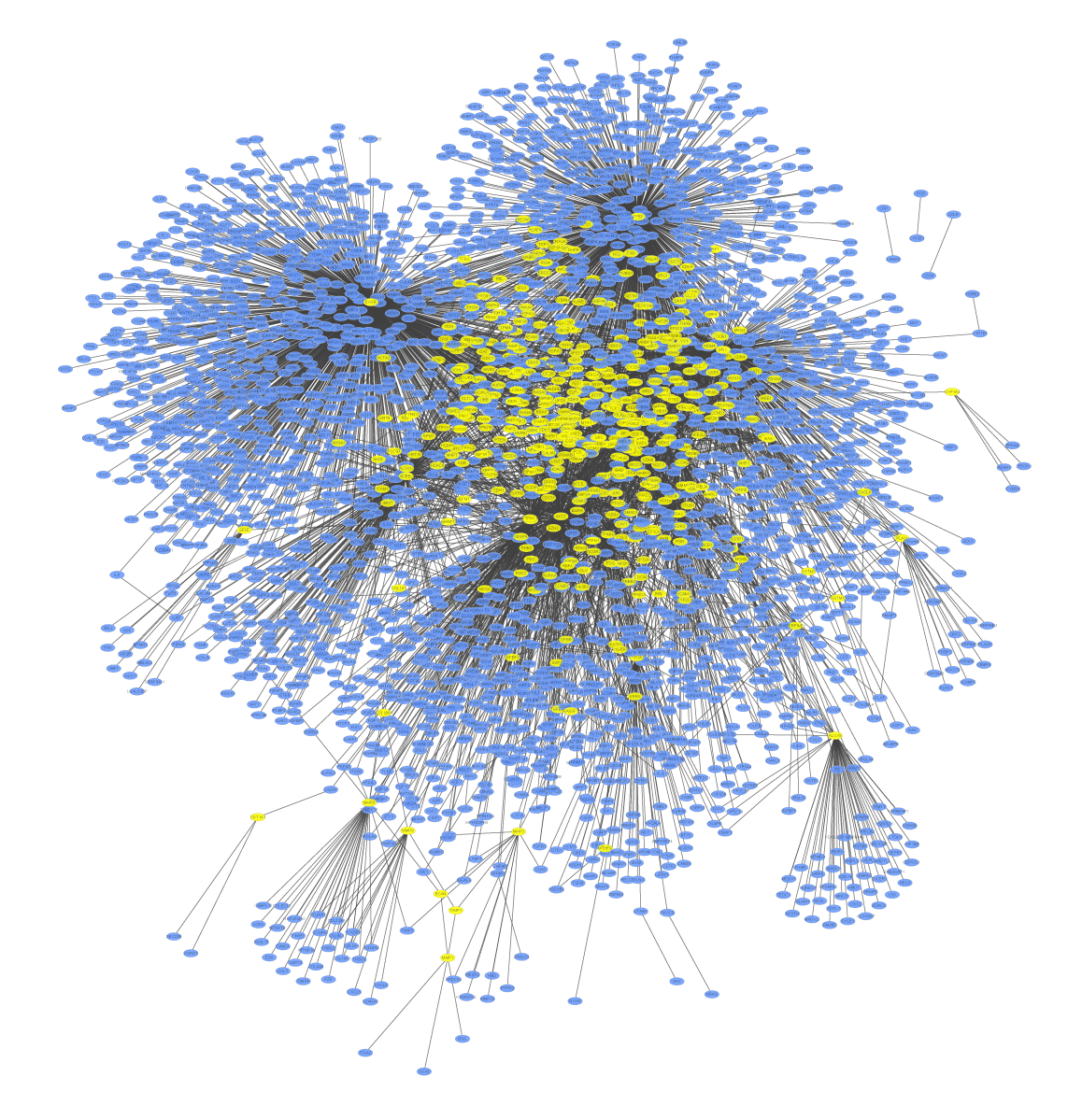

Supplement: Supplementary file 4 — The interactive PPI network of GQD putative targets and known colon cancer-related targets [file 41419_2019_1638_MOESM4_ESM.tif]

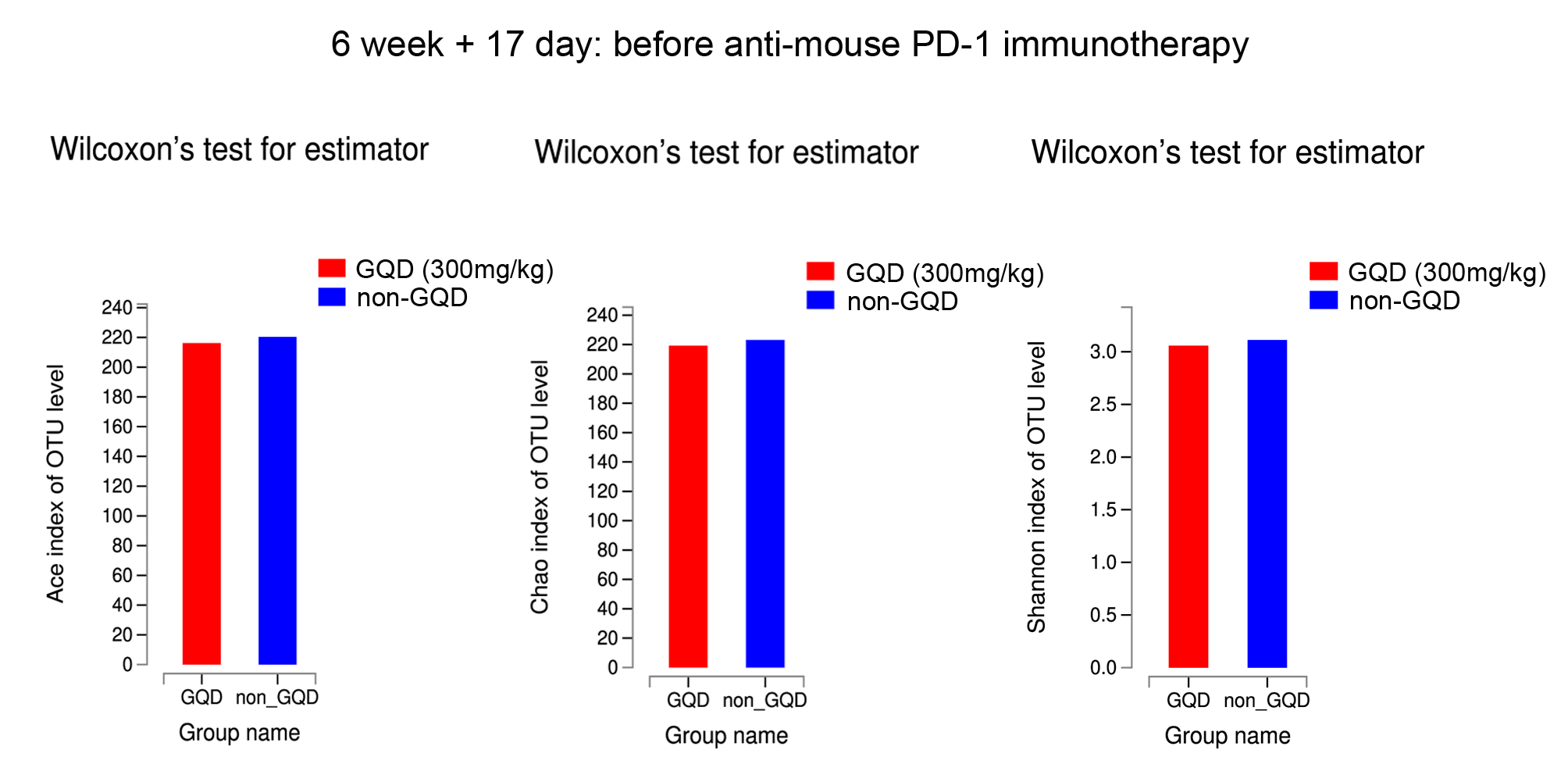

Supplement: Supplementary file 5 — Alpha diversity of the faecal microbiome before anti-mouse PD-1 immunotherapy [file 41419_2019_1638_MOESM5_ESM.tif]

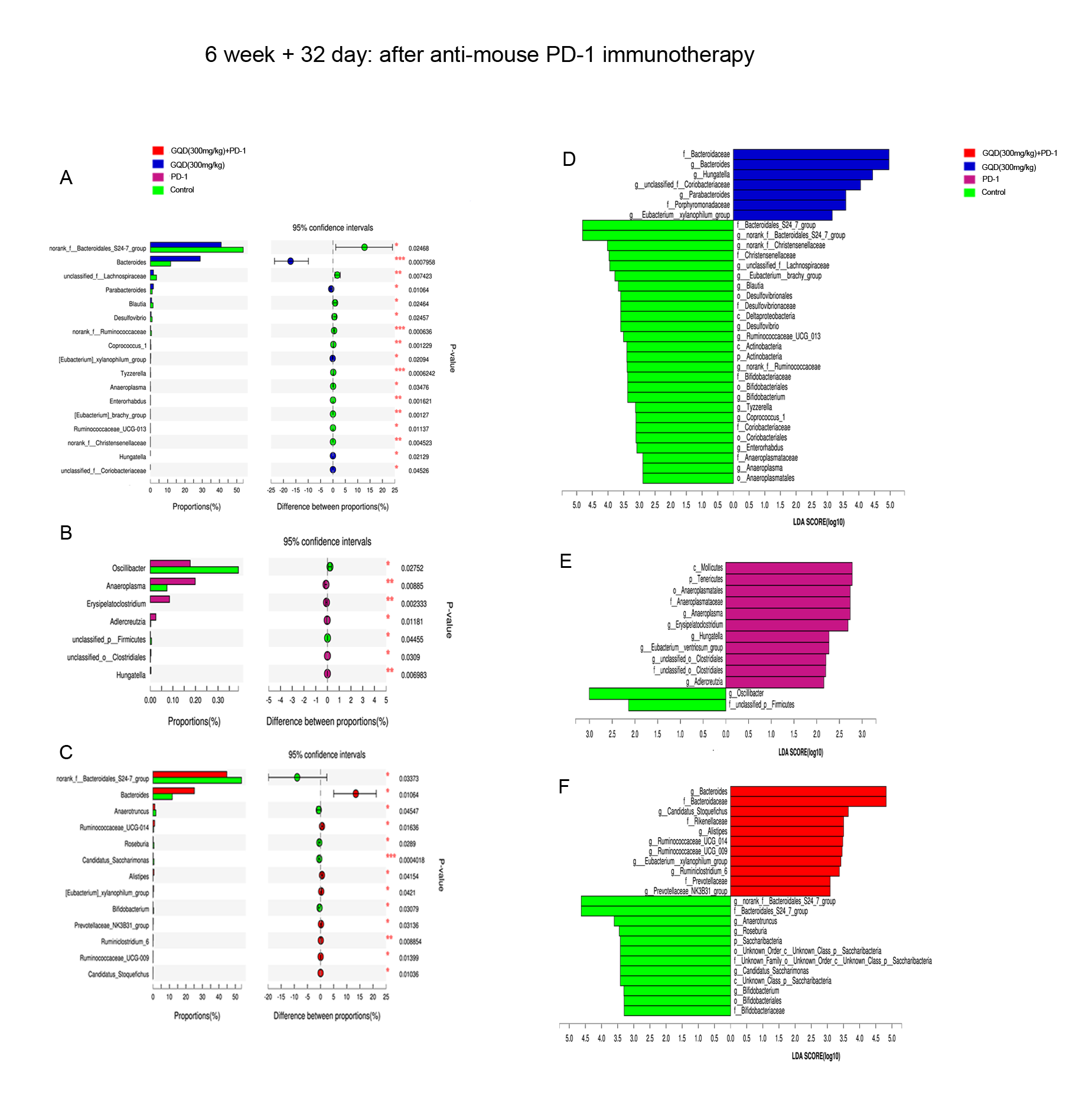

Supplement: Supplementary file 6 — (A, B, C) Bar plot of compositional differences at the genus level in the gut microbiome of mice from each group. (D, E, F) LDA scores computed for differentially- abundant taxa in the faecal mic [file 41419_2019_1638_MOESM6_ESM.tif]

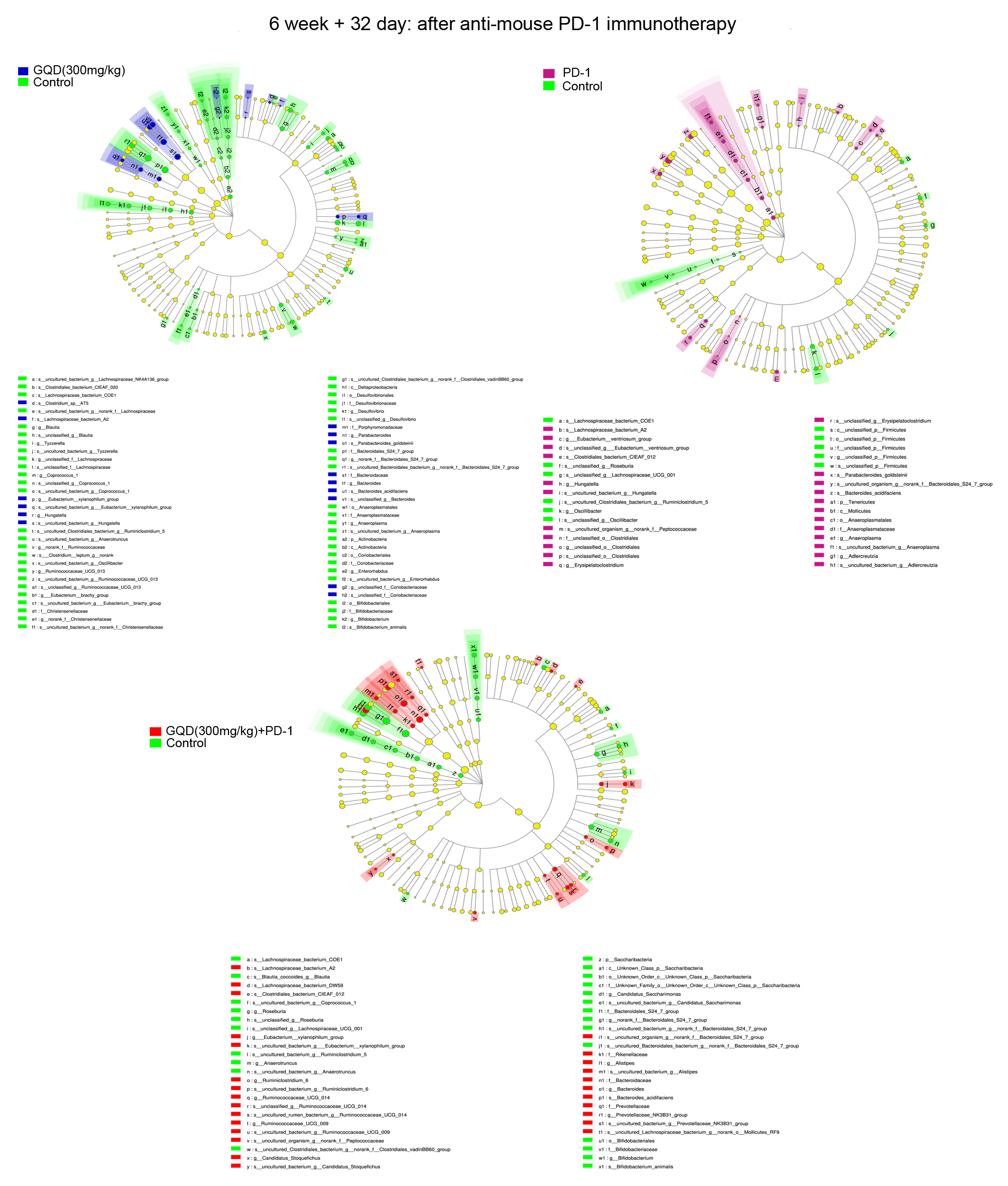

Supplement: Supplementary file 7 — Taxonomic cladogram from LEfSe showing differences in faecal taxa. Dot size is proportional to the abundance of the taxon [file 41419_2019_1638_MOESM7_ESM.tif]

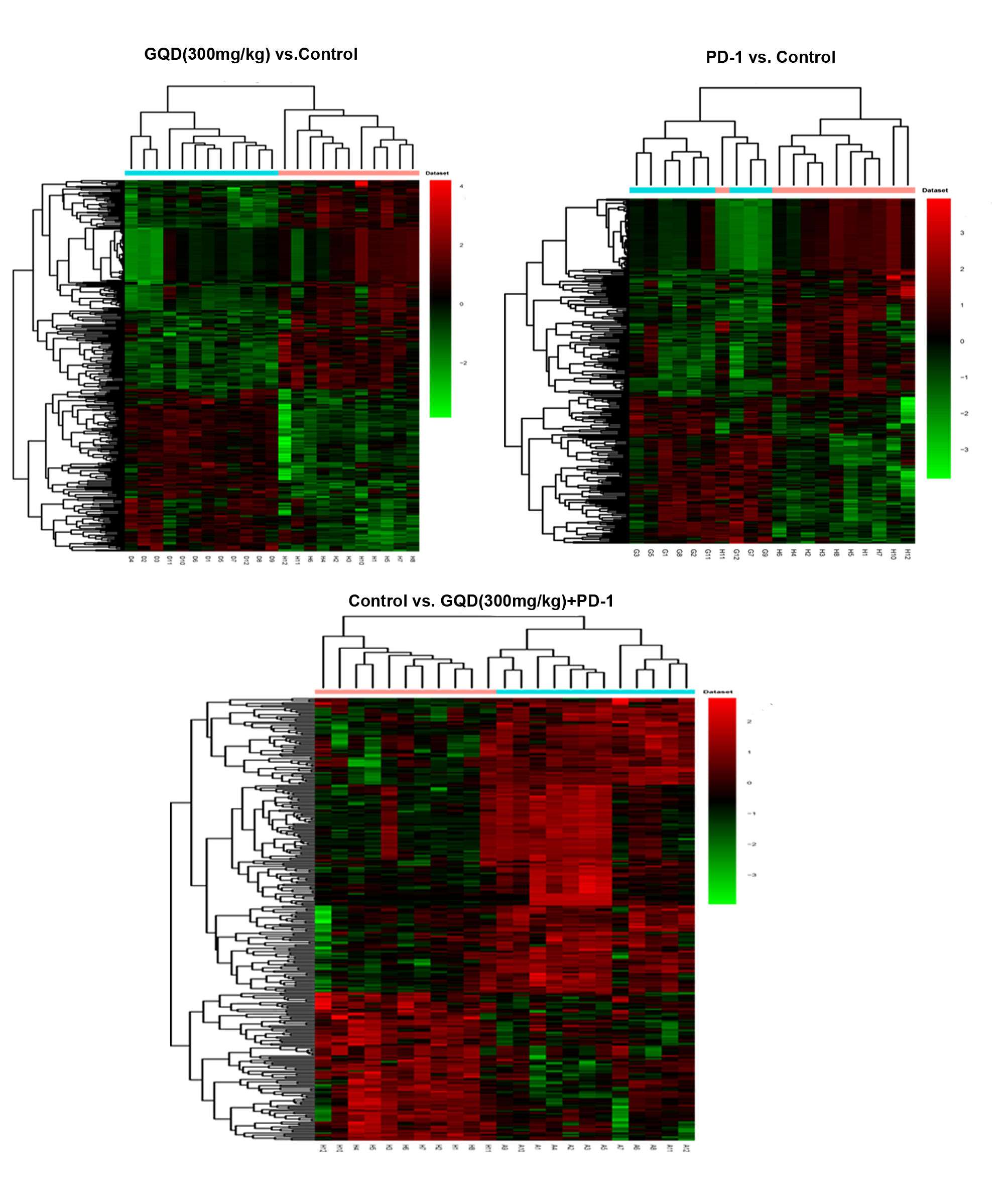

Supplement: Supplementary file 8 — Unsupervised heatmaps of metabolites significantly changed in the GQD vs. control, PD-1 vs. control and GQD+ PD-1 vs. control groups [file 41419_2019_1638_MOESM8_ESM.tif]
