# Supplementary material for: Gegen Qinlian decoction enhances the effect of PD-1 blockade in colorectal cancer with microsatellite stability by remodelling the gut microbiota and the tumour microenvironment
Source: Cell Death Dis. 2019 May 28;10(6):415. doi: 10.1038/s41419-019-1638-6 (PMC6538740; doi:10.1038/s41419-019-1638-6)
Supplement: Supplementary file 9 — Monitoring of precursor-to-product ion pairs, declustering potential (DP) and collision energy (CE) of analytes [file 41419_2019_1638_MOESM9_ESM.docx]

Supplementary Table S1: Monitored precursor-to-product ion pairs, declustering potential (DP) and collision energy (CE) of analytes

| Analyte | MS1(m/z) | MS2(m/z) | DP(V) | CE(eV) |
| --- | --- | --- | --- | --- |
| puerarin | 417.3 | 297.2 | 63 | 36 |
| daidzin | 417.3 | 255.2 | 60 | 23 |
| liquiritin | 436.4 | 257.2 | 24 | 21 |
| baicalin | 447.3 | 271.2 | 95 | 29 |
| berberine | 336.3 | 292.2 | 10 | 20 |
| wogonoside | 461.2 | 285.2 | 89 | 28 |
