# Supplementary material for: Gegen Qinlian decoction enhances the effect of PD-1 blockade in colorectal cancer with microsatellite stability by remodelling the gut microbiota and the tumour microenvironment
Source: Cell Death Dis. 2019 May 28;10(6):415. doi: 10.1038/s41419-019-1638-6 (PMC6538740; doi:10.1038/s41419-019-1638-6)
Supplement: Supplementary file 10 — Known colon cancer-related targets [file 41419_2019_1638_MOESM10_ESM.docx]

Supplementary Table S2: Known colon cancer-related targets

| **Target Gene Name** | **Target Protein Name** | **Source** |
| --- | --- | --- |
| CDH1 | Cadherin-1 | GAD |
| RHPN2 | Rhophilin-2 | GAD |
| SCG5 | Neuroendocrine protein 7B2 | GAD |
| DUSP10 | Dual specificity protein phosphatase 10 | GAD |
| COLCA2 | Colorectal cancer-associated protein 2 | GAD |
| EIF3H | Eukaryotic translation initiation factor 3 subunit H | GAD |
| MYC | Myc proto-oncogene protein | GAD |
| CYP17A1 | Steroid 17-alpha-hydroxylase/17,20 lyase | GAD |
| LAMA5 | Laminin subunit alpha-5 | GAD |
| DIP2B | Disco-interacting protein 2 homolog B | GAD |
| BMP4 | Bone morphogenetic protein 4 | GAD |
| MGST1 | Microsomal glutathione S-transferase 1 | GAD |
| SLC22A3 | Solute carrier family 22 member 3 | GAD |
| MYNN | Myoneurin | GAD |
| TBX3 | T-box transcription factor TBX3 | GAD |
| MTHFR | Methylenetetrahydrofolate reductase | GAD |
| SULT1A1 | Sulfotransferase 1A1 | GAD |
| MMP1 | Interstitial collagenase | GAD |
| CYP2C9 | Cytochrome P450 2C9 | GAD |
| MMP2 | 72 kDa type IV collagenase | GAD |
| HFE | Hereditary hemochromatosis protein | GAD |
| MMP3 | Stromelysin-1 | GAD |
| PTGS2 | Prostaglandin G/H synthase 2 | GAD |
| UGT1A7 | UDP-glucuronosyltransferase 1-7 | GAD |
| XRCC1 | DNA repair protein XRCC1 | GAD |
| CYP1A1 | Cytochrome P450 1A1 | GAD |
| IL6 | Interleukin-6 | GAD |
| ALOX12 | Arachidonate 12-lipoxygenase, 12S-type | GAD |
| PLD2 | Phospholipase D2 | GAD |
| ALOX5 | Arachidonate 5-lipoxygenase | GAD |
| IL8 | Interleukin-8 | GAD |
| CYP1A2 | Cytochrome P450 1A2 | GAD |
| ADRB3 | Beta-3 adrenergic receptor | GAD |
| MDM2 | E3 ubiquitin-protein ligase Mdm2 | GAD |
| NAT2 | Arylamine N-acetyltransferase 2 | GAD |
| CD36 | Platelet glycoprotein 4 | GAD |
| UGT1A6 | UDP-glucuronosyltransferase 1-6 | GAD |
| TRPS1 | Zinc finger transcription factor Trps1 | GAD |
| PPARG | Peroxisome proliferator-activated receptor gamma | GAD |
| GSTT1 | Glutathione S-transferase theta-1 | GAD |
| CYP1B1 | Cytochrome P450 1B1 | GAD |
| VDR | Vitamin D3 receptor | GAD |
| PPARD | Peroxisome proliferator-activated receptor delta | GAD |
| TLR4 | Toll-like receptor 4 | GAD |
| SERPINE1 | Plasminogen activator inhibitor 1 | GAD |
| GSTA1 | Glutathione S-transferase A1 | GAD |
| IGF1 | Insulin-like growth factor I | GAD |
| PLAU | Urokinase-type plasminogen activator | GAD |
| KDR | Vascular endothelial growth factor receptor 2 | GAD |
| CTSB | Cathepsin B | GAD |
| SOD2 | Superoxide dismutase [Mn], mitochondrial | GAD |
| UGT1A1 | UDP-glucuronosyltransferase 1-1 | GAD |
| XRCC3 | DNA repair protein XRCC3 | GAD |
| CYP2D6 | Cytochrome P450 2D6 | GAD |
| GSTM1 | Glutathione S-transferase Mu 1 | GAD |
| FRZB | Secreted frizzled-related protein 3 | GAD |
| ARL11 | ADP-ribosylation factor-like protein 11 | GAD |
| MYCL | Protein L-Myc | GAD |
| GH1 | Somatotropin | GAD |
| LCT | Lactase-phlorizin hydrolase | GAD |
| MTRR | Methionine synthase reductase | GAD |
| NQO1 | NAD(P)H dehydrogenase [quinone] 1 | GAD |
| TGFBR1 | TGF-beta receptor type-1 | GAD |
| CYP2E1 | Cytochrome P450 2E1 | GAD |
| CTLA4 | Cytotoxic T-lymphocyte protein 4 | GAD |
| NFKBIA | NF-kappa-B inhibitor alpha | GAD |
| IGFBP3 | Insulin-like growth factor-binding protein 3 | GAD |
| GSTT2 | Glutathione S-transferase theta-2 | GAD |
| TYMS | Thymidylate synthase | GAD |
| IRS2 | Insulin receptor substrate 2 | GAD |
| RETN | Resistin | GAD |
| CHEK1 | Serine/threonine-protein kinase Chk1 | GAD |
| NOD2 | Nucleotide-binding oligomerization domain-containing protein 2 | GAD |
| ADRB2 | Beta-2 adrenergic receptor | GAD |
| VEGFA | Vascular endothelial growth factor A | GAD |
| MDK | Midkine | GAD |
| EPHX1 | Epoxide hydrolase 1 | GAD |
| IRS1 | Insulin receptor substrate 1 | GAD |
| NAT1 | Arylamine N-acetyltransferase 1 | GAD |
| SLC10A2 | Ileal sodium/bile acid cotransporter | GAD |
| NME1 | Nucleoside diphosphate kinase A | GAD |
| MGMT | Methylated-DNA--protein-cysteine methyltransferase | GAD |
| CHEK2 | Serine/threonine-protein kinase Chk2 | GAD |
| HIF1A | Hypoxia-inducible factor 1-alpha | GAD |
| NOS3 | Nitric oxide synthase, endothelial | GAD |
| XPC | DNA repair protein complementing XP-C cells | GAD |
| GSTP1 | Glutathione S-transferase P | GAD |
| CYP7A1 | Cholesterol 7-alpha-monooxygenase | GAD |
| GSTM3 | Glutathione S-transferase Mu 3 | GAD |
| OGG1 | N-glycosylase/DNA lyase [Includes: 8-oxoguanine DNA glycosylase | GAD |
| CASR | Extracellular calcium-sensing receptor | GAD |
| SELE | E-selectin | GAD |
| ERCC2 | General transcription and DNA repair factor IIH helicase subunit XPD | GAD |
| MTR | Methionine synthase | GAD |
| MMP9 | Matrix metalloproteinase-9 | GAD |
| CD14 | Monocyte differentiation antigen CD14 | GAD |
| MLH1 | DNA mismatch repair protein Mlh1 | GAD; OMIM |
| MSH2 | DNA mismatch repair protein Msh2 | GAD; OMIM |
| APC | Adenomatous polyposis coli protein | GAD; OMIM |
| TGFBR2 | TGF-beta receptor type-2 | GAD; OMIM |
| PIK3CA | Phosphatidylinositol 4,5-bisphosphate 3-kinase catalytic subunit alpha isoform | GAD; OMIM |
| SMAD7 | Mothers against decapentaplegic homolog 7 | GAD; OMIM |
| BRAF | Serine/threonine-protein kinase B-raf | GAD; OMIM |
| TP53 | Cellular tumor antigen p53 | GAD; OMIM |
| CCND1 | G1/S-specific cyclin-D1 | GAD; OMIM |
| ODC1 | Ornithine decarboxylase | GAD; OMIM |
| TLR2 | Toll-like receptor 2 | GAD; OMIM |
| AURKA | Aurora kinase A | GAD; OMIM |
| KRAS | GTPase KRas | GAD; OMIM |
| MUTYH | Adenine DNA glycosylase | GAD; OMIM |
| MSH6 | DNA mismatch repair protein Msh6 | OMIM |
| PMS2 | Mismatch repair endonuclease PMS2 | OMIM |
| MLH3 | DNA mismatch repair protein Mlh3 | OMIM |
| EPCAM | Epithelial cell adhesion molecule | OMIM |
| AXIN2 | Axin-2 | OMIM |
| BMPR1A | Bone morphogenetic protein receptor type-1A | OMIM |
| PMS1 | PMS1 protein homolog 1 | OMIM |
| CTNNB1 | Catenin beta-1 | OMIM |
| NRAS | GTPase NRas | OMIM |
| FLCN | Folliculin | OMIM |
| AKT1 | RAC-alpha serine/threonine-protein kinase | OMIM |
| PLA2G2A | Phospholipase A2, membrane associated | OMIM |
| POLD1 | DNA polymerase delta catalytic subunit | OMIM |
| DCC | Netrin receptor DCC | OMIM |
| BUB1 | Mitotic checkpoint serine/threonine-protein kinase BUB1 | OMIM |
| FGFR3 | Fibroblast growth factor receptor 3 | OMIM |
| BAX | Apoptosis regulator BAX | OMIM |
| MCC | Colorectal mutant cancer protein | OMIM |
| SRC | Proto-oncogene tyrosine-protein kinase Src | OMIM |
| BUB1B | Mitotic checkpoint serine/threonine-protein kinase BUB1 beta | OMIM |
| POLE | DNA polymerase epsilon catalytic subunit A | OMIM |
| GREM1 | Gremlin-1 | OMIM |
| EP300 | Histone acetyltransferase p300 | OMIM |
| PDGFRL | Platelet-derived growth factor receptor-like protein | OMIM |
| PTPN12 | Tyrosine-protein phosphatase non-receptor type 12 | OMIM |
| PTPRJ | Receptor-type tyrosine-protein phosphatase eta | OMIM |
| RAD54B | DNA repair and recombination protein RAD54B | OMIM |
| DLC1 | Rho GTPase-activating protein 7 | OMIM |
| GALNT12 | Polypeptide N-acetylgalactosaminyltransferase 12 | OMIM |
| SOS1 | Son of sevenless homolog 1 | OMIM |
| RAF1 | RAF proto-oncogene serine/threonine-protein kinase | OMIM |
| PTPN11 | Tyrosine-protein phosphatase non-receptor type 11 | OMIM |
| MAP2K1 | Dual specificity mitogen-activated protein kinase kinase 1 | OMIM |
| ALPL | Alkaline phosphatase, tissue-nonspecific isozyme | TTD |
| FLT3 | Receptor-type tyrosine-protein kinase FLT3 | TTD |
| PRKCG | Protein kinase C gamma type | TTD |
| SIRT1 | NAD-dependent protein deacetylase sirtuin-1 | TTD |
| TMP1 | Thymidylate synthase | TTD |
| AKT3 | RAC-gamma serine/threonine-protein kinase | TTD |
| EGFR | Epidermal growth factor receptor | TTD；GAD |
