# Supplementary material for: Gegen Qinlian decoction enhances the effect of PD-1 blockade in colorectal cancer with microsatellite stability by remodelling the gut microbiota and the tumour microenvironment
Source: Cell Death Dis. 2019 May 28;10(6):415. doi: 10.1038/s41419-019-1638-6 (PMC6538740; doi:10.1038/s41419-019-1638-6)
Supplement: Supplementary file 11 — Linear regression data and contents of analytes [file 41419_2019_1638_MOESM11_ESM.docx]

Supplementary Table S3: Linear regression data and contents of analytes

| Analyte | Regression equation | r | Linearity range  (μg/mL) | Content  (mg/g) | RSD(%) |
| --- | --- | --- | --- | --- | --- |
| puerarin | Y = 1.44e^4^ X + 49.2 | r = 0.9949 | 3.144 - 100.6 | 58.4 | 2.5 |
| daidzin | Y = 7.97e^3^ X + 1.4e^4^ | r = 0.9915 | 0.5920 - 18.94 | 62.6 | 2.2 |
| liquiritin | Y = 2.33e^4^ X - 265 | r = 0.9992 | 0.1600 - 5.120 | 2.4 | 4.1 |
| baicalin | Y = 9.86e^3^ X + 6.03e^3^ | r = 0.9915 | 5.786 - 185.2 | 86.8 | 2.4 |
| berberine | Y = 1.07e^5^ X + 9.09e^4^ | r = 0.9904 | 1.440 - 23.04 | 31.5 | 2.5 |
| wogonoside | Y = 3.25e^4^ X - 2.35e^3^ | r = 0.9969 | 1.054 - 33.73 | 14.6 | 2.8 |
