# Supplementary material for: Gegen Qinlian decoction enhances the effect of PD-1 blockade in colorectal cancer with microsatellite stability by remodelling the gut microbiota and the tumour microenvironment
Source: Cell Death Dis. 2019 May 28;10(6):415. doi: 10.1038/s41419-019-1638-6 (PMC6538740; doi:10.1038/s41419-019-1638-6)
Supplement: Supplementary file 12 — Compositive compounds of each compound in GQD [file 41419_2019_1638_MOESM12_ESM.docx]

Supplemental Table S4 : Compositive compounds of each ingredient in GQD

| Herbname | Mol ID | Molecule Name | OB (%) | DL |
| --- | --- | --- | --- | --- |
| *Radix Puerariae* | MOL000392 | formononetin | 69.67 | 0.21 |
| *Radix Puerariae* | MOL000357 | Sitogluside | 20.63 | 0.62 |
| *Radix Puerariae* | MOL000358 | beta-sitosterol | 36.91 | 0.75 |
| *Radix Puerariae* | MOL000390 | daidzein | 19.44 | 0.19 |
| *Radix Puerariae* | MOL000391 | Ononin | 11.52 | 0.78 |
| *Radix Puerariae* | MOL000399 | Docosanoate | 15.69 | 0.26 |
| *Radix Puerariae* | MOL000441 | LUPENONE | 11.66 | 0.78 |
| *Radix Puerariae* | MOL000481 | genistein | 17.93 | 0.21 |
| *Radix Puerariae* | MOL000663 | lignoceric acid | 14.9 | 0.33 |
| *Radix Puerariae* | MOL001999 | scoparone | 74.75 | 0.09 |
| *Radix Puerariae* | MOL002347 | (R)-Allantoin | 96.9 | 0.03 |
| *Radix Puerariae* | MOL002959 | 3'-Methoxydaidzein | 48.57 | 0.24 |
| *Radix Puerariae* | MOL003629 | Daidzein-4,7-diglucoside | 47.27 | 0.67 |
| *Radix Puerariae* | MOL003641 | Soyasapogenol B | 16.73 | 0.75 |
| *Radix Puerariae* | MOL012297 | puerarin | 24.03 | 0.69 |
| *Radix Puerariae* | MOL004631 | 7,8,4'-Trihydroxyisoflavone | 20.67 | 0.22 |
| *Radix Puerariae* | MOL009720 | daidzin | 14.32 | 0.73 |
| *Radix Puerariae* | MOL011797 | (3S,4aR,6aR,6bS,8aR,9R,12aS,14aR,14bR)-4,4,6a,6b,8a,11,11,14b-octamethyl-1,2,3,4a,5,6,7,8,9,10,12,12a,14,14a-tetradecahydropicene-3,9-diol | 17.42 | 0.76 |
| *Scutellariae Radix* | MOL000122 | 1,8-cineole | 39.73 | 0.05 |
| *Scutellariae Radix* | MOL001300 | PEL | 44.03 | 0.02 |
| *Scutellariae Radix* | MOL001689 | acacetin | 34.97 | 0.24 |
| *Scutellariae Radix* | MOL000173 | wogonin | 30.68 | 0.23 |
| *Scutellariae Radix* | MOL013068 | Oroxindin | 7.07 | 0.77 |
| *Scutellariae Radix* | MOL000018 | (+/-)-Isoborneol | 86.98 | 0.05 |
| *Scutellariae Radix* | MOL000198 | (R)-linalool | 39.8 | 0.02 |
| *Scutellariae Radix* | MOL000219 | BOX | 31.55 | 0.02 |
| *Scutellariae Radix* | MOL000228 | (2R)-7-hydroxy-5-methoxy-2-phenylchroman-4-one | 55.23 | 0.2 |
| *Scutellariae Radix* | MOL000024 | alpha-humulene | 22.98 | 0.06 |
| *Scutellariae Radix* | MOL000254 | eugenol | 56.24 | 0.04 |
| *Scutellariae Radix* | MOL002560 | chrysin | 22.61 | 0.18 |
| *Scutellariae Radix* | MOL002573 | β-patchoulene | 50.69 | 0.11 |
| *Scutellariae Radix* | MOL002714 | baicalein | 33.52 | 0.21 |
| *Scutellariae Radix* | MOL002737 | scutellarein | 18.97 | 0.24 |
| *Scutellariae Radix* | MOL002908 | 5,8,2'-Trihydroxy-7-methoxyflavone | 37.01 | 0.27 |
| *Scutellariae Radix* | MOL002909 | 5,7,2,5-tetrahydroxy-8,6-dimethoxyflavone | 33.82 | 0.45 |
| *Scutellariae Radix* | MOL002910 | Carthamidin | 41.15 | 0.24 |
| *Scutellariae Radix* | MOL002911 | 2,6,2',4'-tetrahydroxy-6'-methoxychaleone | 69.04 | 0.22 |
| *Scutellariae Radix* | MOL002912 | Dihydrobaicalin | 20.85 | 0.75 |
| *Scutellariae Radix* | MOL002913 | Dihydrobaicalin_qt | 40.04 | 0.21 |
| *Scutellariae Radix* | MOL002914 | Eriodyctiol (flavanone) | 41.35 | 0.24 |
| *Scutellariae Radix* | MOL002915 | Salvigenin | 49.07 | 0.33 |
| *Scutellariae Radix* | MOL002916 | 2-(2,6-dihydroxyphenyl)-3,5,7-trihydroxy-chromone | 16.44 | 0.27 |
| *Scutellariae Radix* | MOL002917 | 5,2',6'-Trihydroxy-7,8-dimethoxyflavone | 45.05 | 0.33 |
| *Scutellariae Radix* | MOL002918 | Ganhuangenin | 1.34 | 0.37 |
| *Scutellariae Radix* | MOL002919 | Viscidulin III | 14.36 | 0.37 |
| *Scutellariae Radix* | MOL000169 | alpha-Guaiene | 25.93 | 0.07 |
| *Scutellariae Radix* | MOL002921 | (2S,3R,4R,5R,6S)-2-[(2R,3R,4S,5R,6R)-3,5-dihydroxy-2-[2-(3-hydroxy-4-methoxy-phenyl)ethoxy]-6-methylol-tetrahydropyran-4-yl]oxy-6-methyl-tetrahydropyran-3,4,5-triol | 12.69 | 0.67 |
| *Scutellariae Radix* | MOL002922 | 5-(2-hydroxyethyl)-2-methoxyphenol | 31.95 | 0.04 |
| *Scutellariae Radix* | MOL002923 | darendoside B | 10.75 | 0.59 |
| *Scutellariae Radix* | MOL002924 | darendoside B_qt | 10.05 | 0.22 |
| *Scutellariae Radix* | MOL002925 | 5,7,2',6'-Tetrahydroxyflavone | 37.01 | 0.24 |
| *Scutellariae Radix* | MOL002926 | dihydrooroxylin A | 38.72 | 0.23 |
| *Scutellariae Radix* | MOL002927 | Skullcapflavone II | 69.51 | 0.44 |
| *Scutellariae Radix* | MOL002928 | oroxylin a | 41.37 | 0.23 |
| *Scutellariae Radix* | MOL002929 | salidroside | 7.01 | 0.2 |
| *Scutellariae Radix* | MOL002930 | Tyrosol | 33.81 | 0.02 |
| *Scutellariae Radix* | MOL002931 | scutellarin | 2.64 | 0.79 |
| *Scutellariae Radix* | MOL002932 | Panicolin | 76.26 | 0.29 |
| *Scutellariae Radix* | MOL002933 | 5,7,4'-Trihydroxy-8-methoxyflavone | 36.56 | 0.27 |
| *Scutellariae Radix* | MOL002934 | NEOBAICALEIN | 104.34 | 0.44 |
| *Scutellariae Radix* | MOL002935 | Baicalin | 29.53 | 0.77 |
| *Scutellariae Radix* | MOL002936 | 5,8-Dihydroxy-6,7-dimethoxyflavone | 5.74 | 0.29 |
| *Scutellariae Radix* | MOL002937 | DIHYDROOROXYLIN | 66.06 | 0.23 |
| *Scutellariae Radix* | MOL000357 | Sitogluside | 20.63 | 0.62 |
| *Scutellariae Radix* | MOL000358 | beta-sitosterol | 36.91 | 0.75 |
| *Scutellariae Radix* | MOL000359 | sitosterol | 36.91 | 0.75 |
| *Scutellariae Radix* | MOL000396 | (+)-Syringaresinol | 3.29 | 0.72 |
| *Scutellariae Radix* | MOL000458 | campesterol | 5.57 | 0.72 |
| *Scutellariae Radix* | MOL000525 | Norwogonin | 39.4 | 0.21 |
| *Scutellariae Radix* | MOL000552 | 5,2'-Dihydroxy-6,7,8-trimethoxyflavone | 31.71 | 0.35 |
| *Scutellariae Radix* | MOL000612 | (-)-alpha-cedrene | 55.56 | 0.1 |
| *Scutellariae Radix* | MOL000007 | Cosmetin | 9.68 | 0.74 |
| *Scutellariae Radix* | MOL000709 | (S)-Matsutake alcohol | 40.11 | 0.01 |
| *Scutellariae Radix* | MOL000715 | l-Menthone | 57.9 | 0.03 |
| *Scutellariae Radix* | MOL000008 | apigenin | 23.06 | 0.21 |
| *Scutellariae Radix* | MOL003127 | Germacrene D | 19.22 | 0.06 |
| *Scutellariae Radix* | MOL000035 | beta-Selinene | 24.39 | 0.08 |
| *Scutellariae Radix* | MOL000069 | palmitic acid | 19.3 | 0.1 |
| *Scutellariae Radix* | MOL000073 | ent-Epicatechin | 48.96 | 0.24 |
| *Scutellariae Radix* | MOL000131 | EIC | 41.9 | 0.14 |
| *Scutellariae Radix* | MOL000303 | caprylic acid | 16.4 | 0.02 |
| *Scutellariae Radix* | MOL000449 | Stigmasterol | 43.83 | 0.76 |
| *Scutellariae Radix* | MOL000610 | TRD | 17.89 | 0.03 |
| *Scutellariae Radix* | MOL000654 | Methyl montanate | 13.2 | 0.48 |
| *Scutellariae Radix* | MOL000669 | (S)-camphor | 21.68 | 0.05 |
| *Scutellariae Radix* | MOL000676 | DBP | 64.54 | 0.13 |
| *Scutellariae Radix* | MOL000708 | WLN: VHR | 32.63 | 0.01 |
| *Scutellariae Radix* | MOL000714 | Hyacinthin | 38.65 | 0.02 |
| *Scutellariae Radix* | MOL000717 | d-isomenthone | 61.2 | 0.03 |
| *Scutellariae Radix* | MOL000771 | p-coumaric acid | 43.29 | 0.04 |
| *Scutellariae Radix* | MOL000789 | jatrorrizine | 19.65 | 0.59 |
| *Scutellariae Radix* | MOL000860 | stearic acid | 17.83 | 0.14 |
| *Scutellariae Radix* | MOL000864 | MYS | 13.98 | 0.05 |
| *Scutellariae Radix* | MOL000867 | Heptadekan | 8.64 | 0.07 |
| *Scutellariae Radix* | MOL000868 | LFA | 8.46 | 0.13 |
| *Scutellariae Radix* | MOL000869 | Henicosane | 8.41 | 0.15 |
| *Scutellariae Radix* | MOL000870 | HEXATRIACONTANE | 7.95 | 0.41 |
| *Scutellariae Radix* | MOL000879 | methyl palmitate | 18.09 | 0.12 |
| *Scutellariae Radix* | MOL000885 | Dodekan | 17.74 | 0.02 |
| *Scutellariae Radix* | MOL001132 | longipinene | 17.01 | 0.12 |
| *Scutellariae Radix* | MOL001386 | Methyl laurate | 21.75 | 0.05 |
| *Scutellariae Radix* | MOL001392 | Methyl myristate | 19.68 | 0.08 |
| *Scutellariae Radix* | MOL001393 | myristic acid | 21.18 | 0.07 |
| *Scutellariae Radix* | MOL001458 | coptisine | 30.67 | 0.86 |
| *Scutellariae Radix* | MOL001490 | bis[(2S)-2-ethylhexyl] benzene-1,2-dicarboxylate | 43.59 | 0.35 |
| *Scutellariae Radix* | MOL001506 | Supraene | 33.55 | 0.42 |
| *Scutellariae Radix* | MOL001578 | Hypnon | 48.19 | 0.02 |
| *Scutellariae Radix* | MOL001817 | Methyl stearate | 16.8 | 0.16 |
| *Scutellariae Radix* | MOL001818 | Methyl palmitelaidate | 34.61 | 0.12 |
| *Scutellariae Radix* | MOL001889 | Methyl linolelaidate | 41.93 | 0.17 |
| *Scutellariae Radix* | MOL001972 | Pulegone | 51.6 | 0.03 |
| *Scutellariae Radix* | MOL002027 | Methyl behenate | 14.96 | 0.29 |
| *Scutellariae Radix* | MOL002046 | hexanoic acid | 73.08 | 0.01 |
| *Scutellariae Radix* | MOL002137 | OCT | 29.72 | 0.01 |
| *Scutellariae Radix* | MOL002202 | tetramethylpyrazine | 20.01 | 0.03 |
| *Scutellariae Radix* | MOL002378 | UND | 17.15 | 0.02 |
| *Scutellariae Radix* | MOL002819 | catalpol | 5.07 | 0.44 |
| *Scutellariae Radix* | MOL002879 | Diop | 43.59 | 0.39 |
| *Scutellariae Radix* | MOL002897 | epiberberine | 43.09 | 0.78 |
| *Scutellariae Radix* | MOL003050 | nonanoic acid | 40.51 | 0.02 |
| *Scutellariae Radix* | MOL003055 | heptadecyloxirane | 12.44 | 0.15 |
| *Scutellariae Radix* | MOL003393 | (1S,4S)-7-isopropylidene-1,4-dimethyl-2,3,4,5,6,8-hexahydro-1H-azulene | 24.38 | 0.07 |
| *Scutellariae Radix* | MOL003475 | 9-Cedranone | 67.6 | 0.12 |
| *Scutellariae Radix* | MOL003535 | 1,1,6-trimethyl-2H-naphthalene | 24.94 | 0.06 |
| *Scutellariae Radix* | MOL003568 | Patchoulene | 49.06 | 0.11 |
| *Scutellariae Radix* | MOL003920 | Methyl icosanoate | 15.79 | 0.22 |
| *Scutellariae Radix* | MOL004464 | MEHQ | 43.98 | 0.02 |
| *Scutellariae Radix* | MOL004682 | Methyl octylate | 18.71 | 0.02 |
| *Scutellariae Radix* | MOL004684 | methyl (E)-octadec-2-enoate | 29.84 | 0.17 |
| *Scutellariae Radix* | MOL005021 | Mipax | 57.4 | 0.06 |
| *Scutellariae Radix* | MOL005224 | TETRATETRACONTANE | 7.82 | 0.25 |
| *Scutellariae Radix* | MOL005272 | 13-Tetradecenyl acetate | 36.76 | 0.1 |
| *Scutellariae Radix* | MOL005368 | Methyl tricosanoate | 14.61 | 0.33 |
| *Scutellariae Radix* | MOL005402 | Methyl margarate | 17.41 | 0.14 |
| *Scutellariae Radix* | MOL005577 | undecanal | 22.9 | 0.03 |
| *Scutellariae Radix* | MOL005841 | TBP | 27.76 | 0.06 |
| *Scutellariae Radix* | MOL006219 | Clorius | 45.99 | 0.02 |
| *Scutellariae Radix* | MOL006312 | Azulol | 15.15 | 0.07 |
| *Scutellariae Radix* | MOL006370 | 5-o-caffeoylquinic acid | 19.61 | 0.33 |
| *Scutellariae Radix* | MOL007197 | DFA | 31.13 | 0.05 |
| *Scutellariae Radix* | MOL007792 | Isomartynoside | 13.98 | 0.56 |
| *Scutellariae Radix* | MOL008151 | METHYL NONADECANOATE | 16.27 | 0.19 |
| *Scutellariae Radix* | MOL008206 | Moslosooflavone | 44.09 | 0.25 |
| *Scutellariae Radix* | MOL008595 | methyl henicosanoate | 15.36 | 0.26 |
| *Scutellariae Radix* | MOL008615 | Methyl 9-oxononanoate | 24.02 | 0.04 |
| *Scutellariae Radix* | MOL009520 | 3,8-dimethylundecane | 4.72 | 0.03 |
| *Scutellariae Radix* | MOL009730 | methyl icos-11-enoate | 29.49 | 0.23 |
| *Scutellariae Radix* | MOL009734 | Methyl lignocerate | 14.27 | 0.37 |
| *Scutellariae Radix* | MOL010206 | Methyl isoheptadecanoate | 21.72 | 0.14 |
| *Scutellariae Radix* | MOL010415 | 11,13-Eicosadienoic acid, methyl ester | 39.28 | 0.23 |
| *Scutellariae Radix* | MOL010563 | Methyl (Z)-cinnamate | 37.2 | 0.04 |
| *Scutellariae Radix* | MOL011081 | Linolenic acid methyl ester | 46.15 | 0.17 |
| *Scutellariae Radix* | MOL011322 | Diisobutyl succinate | 39.54 | 0.06 |
| *Scutellariae Radix* | MOL012240 | 2',3',5,7-tetrahydroxyflavone | 25.75 | 0.24 |
| *Scutellariae Radix* | MOL012245 | 5,7,4'-trihydroxy-6-methoxyflavanone | 36.63 | 0.27 |
| *Scutellariae Radix* | MOL012246 | 5,7,4'-trihydroxy-8-methoxyflavanone | 74.24 | 0.26 |
| *Scutellariae Radix* | MOL012266 | rivularin | 37.94 | 0.37 |
| *Scutellariae Radix* | MOL012267 | Scutevulin | 20.67 | 0.27 |
| *Scutellariae Radix* | MOL012564 | 3,7-dimethylnonane | 15.31 | 0.02 |
| *Scutellariae Radix* | MOL013062 | BZQ | 58.62 | 0.06 |
| *Scutellariae Radix* | MOL013161 | METHYL HEXACOSANOATE | 13.68 | 0.43 |
| *Coptidis Rhizoma* | MOL001454 | berberine | 36.86 | 0.78 |
| *Coptidis Rhizoma* | MOL001457 | columbamine | 26.94 | 0.59 |
| *Coptidis Rhizoma* | MOL001867 | Isovanillin | 31.01 | 0.03 |
| *Coptidis Rhizoma* | MOL001955 | Heriguard | 11.93 | 0.33 |
| *Coptidis Rhizoma* | MOL002329 | Javanicin | 17.38 | 0.78 |
| *Coptidis Rhizoma* | MOL002637 | Obacunoic acid | 20.69 | 0.79 |
| *Coptidis Rhizoma* | MOL002639 | Obamegine | 2.55 | 0.11 |
| *Coptidis Rhizoma* | MOL002664 | Fagarine | 72.23 | 0.15 |
| *Coptidis Rhizoma* | MOL002890 | 2-Carboxymethyl-3-prenyl-2,3-epoxy-1,4-naphthoquinone | 20.68 | 0.26 |
| *Coptidis Rhizoma* | MOL002891 | magnoflorine | 0.48 | 0.55 |
| *Coptidis Rhizoma* | MOL013352 | Obacunone | 43.29 | 0.77 |
| *Coptidis Rhizoma* | MOL002893 | Trihydroxybufosterocholanic acid | 16.78 | 0.84 |
| *Coptidis Rhizoma* | MOL002894 | berberrubine | 35.74 | 0.73 |
| *Coptidis Rhizoma* | MOL002895 | DPEC | 28.36 | 0.24 |
| *Coptidis Rhizoma* | MOL002896 | Corydaldine | 49.3 | 0.09 |
| *Coptidis Rhizoma* | MOL002897 | epiberberine | 43.09 | 0.78 |
| *Coptidis Rhizoma* | MOL002898 | groenlandicine | 28.42 | 0.72 |
| *Coptidis Rhizoma* | MOL003959 | limonin | 21.3 | 0.57 |
| *Coptidis Rhizoma* | MOL002900 | Noroxyhydrastinine | 38.89 | 0.1 |
| *Coptidis Rhizoma* | MOL002901 | phellodendrine | 2.5 | 0.58 |
| *Coptidis Rhizoma* | MOL002902 | Ethyl caffeate | 103.85 | 0.07 |
| *Coptidis Rhizoma* | MOL002903 | (R)-Canadine | 55.37 | 0.77 |
| *Coptidis Rhizoma* | MOL002904 | Berlambine | 36.68 | 0.82 |
| *Coptidis Rhizoma* | MOL002905 | Zosimin | 14.82 | 0.36 |
| *Coptidis Rhizoma* | MOL002906 | Corchoroside A | 19.6 | 0.69 |
| *Coptidis Rhizoma* | MOL002907 | Corchoroside A_qt | 104.95 | 0.78 |
| *Coptidis Rhizoma* | MOL000360 | FER | 39.56 | 0.06 |
| *Coptidis Rhizoma* | MOL000622 | Magnograndiolide | 63.71 | 0.19 |
| *Coptidis Rhizoma* | MOL000762 | Palmidin A | 35.36 | 0.65 |
| *Coptidis Rhizoma* | MOL000778 | 6-O-E-Feruloylajugol | 26.13 | 0.85 |
| *Coptidis Rhizoma* | MOL000779 | 6-O-E-Feruloylajugol_qt | 15.46 | 0.43 |
| *Coptidis Rhizoma* | MOL000785 | palmatine | 64.6 | 0.65 |
| *Coptidis Rhizoma* | MOL000789 | jatrorrizine | 19.65 | 0.59 |
| *Coptidis Rhizoma* | MOL000098 | quercetin | 46.43 | 0.28 |
| *Coptidis Rhizoma* | MOL000114 | vanillic acid | 35.47 | 0.04 |
| *Coptidis Rhizoma* | MOL000141 | hydroxytyrosol | 57.57 | 0.03 |
| *Coptidis Rhizoma* | MOL000771 | p-coumaric acid | 43.29 | 0.04 |
| *Coptidis Rhizoma* | MOL001458 | coptisine | 30.67 | 0.86 |
| *Coptidis Rhizoma* | MOL001845 | clemastanin B_qt | 5.53 | 0.38 |
| *Coptidis Rhizoma* | MOL002343 | tetrandrine | 26.64 | 0.1 |
| *Coptidis Rhizoma* | MOL002668 | Worenine | 45.83 | 0.87 |
| *Coptidis Rhizoma* | MOL003178 | GENOP | 29.33 | 0.04 |
| *Coptidis Rhizoma* | MOL003503 | Methyl protocatechuate | 38.19 | 0.04 |
| *Coptidis Rhizoma* | MOL004095 | Cinnamic acid, 3,4-dimethoxy- (8CI) | 63.86 | 0.07 |
| *Coptidis Rhizoma* | MOL006972 | Pycnamine | 27.61 | 0.11 |
| *Coptidis Rhizoma* | MOL007134 | danshensu | 36.91 | 0.06 |
| *Coptidis Rhizoma* | MOL008647 | Moupinamide | 86.71 | 0.26 |
| *Coptidis Rhizoma* | MOL013012 | Oxyacanthine | 9.91 | 0.11 |
| *licorice* | MOL000105 | protocatechuic acid | 25.37 | 0.04 |
| *licorice* | MOL001097 | o-xylene | 45.55 | 0.01 |
| *licorice* | MOL001098 | m-xylene | 47.43 | 0.01 |
| *licorice* | MOL001099 | p-xylene | 48.74 | 0.01 |
| *licorice* | MOL000118 | (L)-alpha-Terpineol | 48.8 | 0.03 |
| *licorice* | MOL000012 | Arachic acid | 16.66 | 0.19 |
| *licorice* | MOL001484 | Inermine | 75.18 | 0.54 |
| *licorice* | MOL001543 | Vicenin-2 | 3.42 | 0.78 |
| *licorice* | MOL001599 | α-cubebol | 64.81 | 0.09 |
| *licorice* | MOL001696 | Morusin | 11.52 | 0.76 |
| *licorice* | MOL001737 | ICO | 33.86 | 0.05 |
| *licorice* | MOL001789 | isoliquiritigenin | 85.32 | 0.15 |
| *licorice* | MOL001792 | DFV | 32.76 | 0.18 |
| *licorice* | MOL001850 | Izoforon | 44.98 | 0.03 |
| *licorice* | MOL000211 | Mairin | 55.38 | 0.78 |
| *licorice* | MOL002137 | OCT | 29.72 | 0.01 |
| *licorice* | MOL002166 | ISOHEPTANE | 59.94 | 0.01 |
| *licorice* | MOL002198 | Heptan | 41.8 | 0 |
| *licorice* | MOL002311 | Glycyrol | 90.78 | 0.67 |
| *licorice* | MOL000239 | Jaranol | 50.83 | 0.29 |
| *licorice* | MOL002547 | 21987_FLUKA | 40.92 | 0.04 |
| *licorice* | MOL002565 | Medicarpin | 49.22 | 0.34 |
| *licorice* | MOL000263 | oleanolic acid | 29.02 | 0.76 |
| *licorice* | MOL002678 | EB | 49.38 | 0.01 |
| *licorice* | MOL002693 | nicotiflorin | 3.64 | 0.73 |
| *licorice* | MOL002844 | Pinocembrin | 64.72 | 0.18 |
| *licorice* | MOL002850 | butylated hydroxytoluene | 40.02 | 0.07 |
| *licorice* | MOL002943 | BuOH | 22.02 | 0 |
| *licorice* | MOL003218 | Neouralenol | 12.76 | 0.46 |
| *licorice* | MOL000354 | isorhamnetin | 49.6 | 0.31 |
| *licorice* | MOL000359 | sitosterol | 36.91 | 0.75 |
| *licorice* | MOL003656 | Lupiwighteone | 51.64 | 0.37 |
| *licorice* | MOL003662 | 7,4'-Dihydroxyflavone | 19.18 | 0.18 |
| *licorice* | MOL003686 | Narcissoside | 5.09 | 0.65 |
| *licorice* | MOL003896 | 7-Methoxy-2-methyl isoflavone | 42.56 | 0.2 |
| *licorice* | MOL000392 | formononetin | 69.67 | 0.21 |
| *licorice* | MOL003985 | 2-Caren-10-al | 44.74 | 0.05 |
| *licorice* | MOL000040 | Scopoletol | 27.77 | 0.08 |
| *licorice* | MOL000415 | rutin | 3.2 | 0.68 |
| *licorice* | MOL000417 | Calycosin | 47.75 | 0.24 |
| *licorice* | MOL000422 | kaempferol | 41.88 | 0.24 |
| *licorice* | MOL004328 | naringenin | 59.29 | 0.21 |
| *licorice* | MOL000437 | Hirsutrin | 1.86 | 0.77 |
| *licorice* | MOL000445 | 8-Prenylwighteone | 23.22 | 0.54 |
| *licorice* | MOL004589 | Methylheptane | 28.65 | 0.01 |
| *licorice* | MOL000467 | Castanin | 23.54 | 0.27 |
| *licorice* | MOL004723 | beta-Terpinene | 42.29 | 0.02 |
| *licorice* | MOL000475 | anethole | 32.49 | 0.03 |
| *licorice* | MOL004801 | 2',7-Dihydroxy-4'-methoxyisoflavan-7-O-β-d-glucopyranoside | 10.46 | 0.73 |
| *licorice* | MOL004802 | (E)-1-butoxyhex-2-ene | 41.72 | 0.02 |
| *licorice* | MOL004803 | 3-Hydroxyglabrol | 4.73 | 0.58 |
| *licorice* | MOL004804 | 18beta-glycyrrhetinic acid | 22.05 | 0.74 |
| *licorice* | MOL004805 | (2S)-2-[4-hydroxy-3-(3-methylbut-2-enyl)phenyl]-8,8-dimethyl-2,3-dihydropyrano[2,3-f]chromen-4-one | 31.79 | 0.72 |
| *licorice* | MOL004806 | euchrenone | 30.29 | 0.57 |
| *licorice* | MOL004807 | glucuronic acid | 46.18 | 0.06 |
| *licorice* | MOL004808 | glyasperin B | 65.22 | 0.44 |
| *licorice* | MOL004809 | glyasperin E | 4.12 | 0.75 |
| *licorice* | MOL004810 | glyasperin F | 75.84 | 0.54 |
| *licorice* | MOL004811 | Glyasperin C | 45.56 | 0.4 |
| *licorice* | MOL004812 | glyasperins D | 29.91 | 0.43 |
| *licorice* | MOL004813 | glyasperins Z | 4.17 | 0.36 |
| *licorice* | MOL004814 | Isotrifoliol | 31.94 | 0.42 |
| *licorice* | MOL004815 | (E)-1-(2,4-dihydroxyphenyl)-3-(2,2-dimethylchromen-6-yl)prop-2-en-1-one | 39.62 | 0.35 |
| *licorice* | MOL004816 | (2R)-1-[2,4-dihydroxy-5-(3-methylbut-2-enyl)phenyl]-2-hydroxy-3-[4-hydroxy-3-(3-methylbut-2-enyl)phenyl]propan-1-one | 1.06 | 0.48 |
| *licorice* | MOL004817 | kanzonols K | 0.97 | 0.66 |
| *licorice* | MOL004818 | kanzonols L | 0.98 | 0.78 |
| *licorice* | MOL004819 | kanzonols T | 17.87 | 0.67 |
| *licorice* | MOL004820 | kanzonols W | 50.48 | 0.52 |
| *licorice* | MOL004821 | kanzonols X | 7.56 | 0.56 |
| *licorice* | MOL004822 | (E)-1-(2,4-dihydroxyphenyl)-3-[4-hydroxy-3-(3-methylbut-2-enyl)phenyl]prop-2-en-1-one | 1.04 | 0.27 |
| *licorice* | MOL004823 | licoagropin | 27.14 | 0.51 |
| *licorice* | MOL004824 | (2S)-6-(2,4-dihydroxyphenyl)-2-(2-hydroxypropan-2-yl)-4-methoxy-2,3-dihydrofuro[3,2-g]chromen-7-one | 60.25 | 0.63 |
| *licorice* | MOL004825 | glyinflanin A | 1.06 | 0.48 |
| *licorice* | MOL005812 | naringin | 6.92 | 0.78 |
| *licorice* | MOL004827 | Semilicoisoflavone B | 48.78 | 0.55 |
| *licorice* | MOL004828 | Glepidotin A | 44.72 | 0.35 |
| *licorice* | MOL004829 | Glepidotin B | 64.46 | 0.34 |
| *licorice* | MOL004830 | Octadiene | 34.53 | 0.01 |
| *licorice* | MOL004831 | (E)-1-[2,4-dihydroxy-3-(3-methylbut-2-enyl)phenyl]-3-[4-hydroxy-3-(3-methylbut-2-enyl)phenyl]prop-2-en-1-one | 1.02 | 0.45 |
| *licorice* | MOL004832 | WLN: 4OVR | 48.41 | 0.04 |
| *licorice* | MOL004833 | Phaseolinisoflavan | 32.01 | 0.45 |
| *licorice* | MOL004834 | 3-(2-hydroxy-4-methoxyphenyl)-2H-chromen-7-ol | 4.66 | 0.21 |
| *licorice* | MOL004835 | Glypallichalcone | 61.6 | 0.19 |
| *licorice* | MOL004836 | echinatin | 66.58 | 0.17 |
| *licorice* | MOL004837 | Karenzu DK2 | 62.26 | 0.1 |
| *licorice* | MOL004838 | 8-(6-hydroxy-2-benzofuranyl)-2,2-dimethyl-5-chromenol | 58.44 | 0.38 |
| *licorice* | MOL004839 | (1S,2S)-1,2-dimethylcyclopentane | 41.78 | 0.01 |
| *licorice* | MOL004840 | Liconeolignan | 4.41 | 0.4 |
| *licorice* | MOL004841 | Licochalcone B | 76.76 | 0.19 |
| *licorice* | MOL004842 | licochalcone C | 4.44 | 0.29 |
| *licorice* | MOL004843 | licochalconeD | 1.01 | 0.34 |
| *licorice* | MOL004844 | glabrol | 4.25 | 0.54 |
| *licorice* | MOL004845 | apioglycyrrhizin | 17.8 | 0.14 |
| *licorice* | MOL004846 | apioglycyrrhizin_qt | 23.73 | 0.74 |
| *licorice* | MOL004847 | 2,2-DIMETHYLPENTANE | 55.33 | 0.01 |
| *licorice* | MOL004848 | licochalcone G | 49.25 | 0.32 |
| *licorice* | MOL004849 | 3-(2,4-dihydroxyphenyl)-8-(1,1-dimethylprop-2-enyl)-7-hydroxy-5-methoxy-coumarin | 59.62 | 0.43 |
| *licorice* | MOL004850 | liquoric acid | 25.44 | 0.55 |
| *licorice* | MOL004851 | Licoflavone | 18.75 | 0.33 |
| *licorice* | MOL004852 | 7-hydroxy-2-[4-hydroxy-3-(3-methylbut-2-enyl)phenyl]-6-(3-methylbut-2-enyl)chromone | 4.44 | 0.56 |
| *licorice* | MOL004853 | Licoflavonol | 8.75 | 0.4 |
| *licorice* | MOL004385 | Yinyanghuo D | 13.99 | 0.38 |
| *licorice* | MOL004855 | Licoricone | 63.58 | 0.47 |
| *licorice* | MOL004856 | Gancaonin A | 51.08 | 0.4 |
| *licorice* | MOL004857 | Gancaonin B | 48.79 | 0.45 |
| *licorice* | MOL004858 | Gancaonin C | 2.87 | 0.42 |
| *licorice* | MOL004859 | 2,3-dimethylhexane | 46.24 | 0.01 |
| *licorice* | MOL000486 | Prunetin | 5.41 | 0.24 |
| *licorice* | MOL004860 | licorice glycoside E | 32.89 | 0.27 |
| *licorice* | MOL004861 | Gancaonin D | 2.72 | 0.51 |
| *licorice* | MOL004862 | (2R)-2-[3,4-dihydroxy-5-(3-methylbut-2-enyl)phenyl]-5,7-dihydroxy-8-(3-methylbut-2-enyl)chroman-4-one | 1.21 | 0.63 |
| *licorice* | MOL004863 | 3-(3,4-dihydroxyphenyl)-5,7-dihydroxy-8-(3-methylbut-2-enyl)chromone | 66.37 | 0.41 |
| *licorice* | MOL004864 | 5,7-dihydroxy-3-(4-methoxyphenyl)-8-(3-methylbut-2-enyl)chromone | 30.49 | 0.41 |
| *licorice* | MOL004865 | 5,7-dihydroxy-3-(2-hydroxy-4-methoxy-phenyl)-6-(3-methylbut-2-enyl)chromone | 2.47 | 0.45 |
| *licorice* | MOL004866 | 2-(3,4-dihydroxyphenyl)-5,7-dihydroxy-6-(3-methylbut-2-enyl)chromone | 44.15 | 0.41 |
| *licorice* | MOL004867 | Gancaonin P | 1.41 | 0.45 |
| *licorice* | MOL004868 | Gancaonin Q | 8.98 | 0.6 |
| *licorice* | MOL004869 | Gancaonin R | 1.26 | 0.37 |
| *licorice* | MOL004870 | Gancaonin S | 1.26 | 0.38 |
| *licorice* | MOL004871 | (3S)-2,3-dimethylpentane | 35.57 | 0.01 |
| *licorice* | MOL004872 | gancaonin T | 1.04 | 0.53 |
| *licorice* | MOL004873 | Gancaonin U | 14.53 | 0.53 |
| *licorice* | MOL004874 | Gancaonin V | 1.24 | 0.34 |
| *licorice* | MOL004875 | 3-[4,6-dihydroxy-2-methoxy-3-(3-methylbut-2-enyl)phenyl]-7-hydroxy-chromone | 2.47 | 0.44 |
| *licorice* | MOL004876 | Glycyram | 19.62 | 0.11 |
| *licorice* | MOL004877 | Licoricidin | 0.99 | 0.62 |
| *licorice* | MOL004878 | Glycycoumarin | 23.56 | 0.44 |
| *licorice* | MOL004879 | Glycyrin | 52.61 | 0.47 |
| *licorice* | MOL004880 | 5,6,7,8-Tetrahydro-2,4-dimethylquinoline | 49.77 | 0.05 |
| *licorice* | MOL004881 | (E)-1-[2,4-dihydroxy-3-(3-methylbut-2-enyl)phenyl]-3-(2,4-dihydroxyphenyl)prop-2-en-1-one | 1.36 | 0.3 |
| *licorice* | MOL004882 | Licocoumarone | 33.21 | 0.36 |
| *licorice* | MOL004883 | Licoisoflavone | 41.61 | 0.42 |
| *licorice* | MOL004884 | Licoisoflavone B | 38.93 | 0.55 |
| *licorice* | MOL004885 | licoisoflavanone | 52.47 | 0.54 |
| *licorice* | MOL004886 | licorice-saponin C2 | 59.66 | 0.11 |
| *licorice* | MOL004887 | licorice-saponin C2_qt | 17.33 | 0.76 |
| *licorice* | MOL004888 | licorice-saponin F3 | 17.68 | 0.03 |
| *licorice* | MOL004889 | licorice-saponin F3_qt | 27.53 | 0.64 |
| *licorice* | MOL004890 | (4S)-2,4-dimethylhexane | 37.13 | 0.01 |
| *licorice* | MOL004891 | shinpterocarpin | 80.3 | 0.73 |
| *licorice* | MOL004892 | licorice-saponin G2 | 6.39 | 0.11 |
| *licorice* | MOL004893 | licorice-saponin G2_qt | 22.78 | 0.72 |
| *licorice* | MOL004894 | licorice-saponin H2 | 44.37 | 0.11 |
| *licorice* | MOL004895 | licorice-saponin H2_qt | 22.91 | 0.74 |
| *licorice* | MOL004896 | licorice-saponin J2 | 6.25 | 0.11 |
| *licorice* | MOL004897 | licorice-saponin J2_qt | 28.3 | 0.74 |
| *licorice* | MOL004898 | (E)-3-[3,4-dihydroxy-5-(3-methylbut-2-enyl)phenyl]-1-(2,4-dihydroxyphenyl)prop-2-en-1-one | 46.27 | 0.31 |
| *licorice* | MOL004899 | licorice-saponin B2 | 58.55 | 0.11 |
| *licorice* | MOL004900 | licorice-saponin K2 | 7.82 | 0.11 |
| *licorice* | MOL004901 | licorice-saponin K2_qt | 27.79 | 0.75 |
| *licorice* | MOL004902 | glycyrrhetol | 14.66 | 0.75 |
| *licorice* | MOL004903 | liquiritin | 65.69 | 0.74 |
| *licorice* | MOL004904 | licopyranocoumarin | 80.36 | 0.65 |
| *licorice* | MOL004905 | 3,22-Dihydroxy-11-oxo-delta(12)-oleanene-27-alpha-methoxycarbonyl-29-oic acid | 34.32 | 0.55 |
| *licorice* | MOL004906 | Hispaglabridin B | 22.94 | 0.88 |
| *licorice* | MOL004907 | Glyzaglabrin | 61.07 | 0.35 |
| *licorice* | MOL004908 | Glabridin | 53.25 | 0.47 |
| *licorice* | MOL004909 | glabrolide | 17.46 | 0.61 |
| *licorice* | MOL004910 | Glabranin | 52.9 | 0.31 |
| *licorice* | MOL004911 | Glabrene | 46.27 | 0.44 |
| *licorice* | MOL004912 | Glabrone | 52.51 | 0.5 |
| *licorice* | MOL004913 | 1,3-dihydroxy-9-methoxy-6-benzofurano[3,2-c]chromenone | 48.14 | 0.43 |
| *licorice* | MOL004914 | 1,3-dihydroxy-8,9-dimethoxy-6-benzofurano[3,2-c]chromenone | 62.9 | 0.53 |
| *licorice* | MOL004915 | Eurycarpin A | 43.28 | 0.37 |
| *licorice* | MOL004916 | 2-methyl-5-propyl -nonane | 15.28 | 0.03 |
| *licorice* | MOL004917 | glycyroside | 37.25 | 0.79 |
| *licorice* | MOL004918 | HEX | 52.5 | 0 |
| *licorice* | MOL004919 | Sextone B | 56.2 | 0.01 |
| *licorice* | MOL004920 | Methylcyclopentane | 55.78 | 0.01 |
| *licorice* | MOL004921 | Docosyl caffeate | 3.14 | 0.59 |
| *licorice* | MOL004922 | 2-methyl-6-ethyl decane | 5.5 | 0.03 |
| *licorice* | MOL000391 | Ononin | 11.52 | 0.78 |
| *licorice* | MOL004924 | (-)-Medicocarpin | 40.99 | 0.95 |
| *licorice* | MOL004925 | vitexin | 3.05 | 0.71 |
| *licorice* | MOL004926 | 4H-1-Benzopyran-4-one, 2-(4-(beta-D-glucopyranosyloxy)phenyl)-2,3-dihydro-5,7-dihydroxy-, (2S)- | 14.03 | 0.78 |
| *licorice* | MOL004927 | Hispaglabridin A | 14.6 | 0.73 |
| *licorice* | MOL004928 | violanthin | 4.17 | 0.81 |
| *licorice* | MOL004929 | Pentadecanol | 13.73 | 0.06 |
| *licorice* | MOL004930 | Uralenol | 8.55 | 0.46 |
| *licorice* | MOL004931 | Uralenol-3-methylether | 1.41 | 0.49 |
| *licorice* | MOL004932 | glycyrrhizin | 9.06 | 0.11 |
| *licorice* | MOL004933 | uralsaponin B | 7.92 | 0.11 |
| *licorice* | MOL004934 | Isohexane | 56.13 | 0 |
| *licorice* | MOL004935 | Sigmoidin-B | 34.88 | 0.41 |
| *licorice* | MOL004936 | Uralene | 11.7 | 0.49 |
| *licorice* | MOL004937 | uralenneoside | 24.96 | 0.17 |
| *licorice* | MOL004938 | schaftoside | 7.88 | 0.75 |
| *licorice* | MOL004939 | Nortangeretin | 17.9 | 0.27 |
| *licorice* | MOL004940 | neoliquiritin | 13.01 | 0.71 |
| *licorice* | MOL004941 | (2R)-7-hydroxy-2-(4-hydroxyphenyl)chroman-4-one | 71.12 | 0.18 |
| *licorice* | MOL004942 | (E)-dodec-2-ene | 17.74 | 0.02 |
| *licorice* | MOL004943 | neoisoliquiritin | 21.18 | 0.58 |
| *licorice* | MOL004944 | Cyclobutanol, 1-ethyl- | 93.23 | 0.02 |
| *licorice* | MOL004945 | (2S)-7-hydroxy-2-(4-hydroxyphenyl)-8-(3-methylbut-2-enyl)chroman-4-one | 36.57 | 0.32 |
| *licorice* | MOL004946 | 2-Tetradecanone | 17.71 | 0.05 |
| *licorice* | MOL004947 | Isoviolanthin | 18.79 | 0.81 |
| *licorice* | MOL004948 | Isoglycyrol | 44.7 | 0.84 |
| *licorice* | MOL004949 | Isolicoflavonol | 45.17 | 0.42 |
| *licorice* | MOL004950 | isoglycycoumarin | 22.09 | 0.6 |
| *licorice* | MOL004951 | Isoliquiritin | 8.61 | 0.6 |
| *licorice* | MOL004952 | licuraside | 5.25 | 0.77 |
| *licorice* | MOL004953 | Liquiritin apioside | 29.23 | 0.82 |
| *licorice* | MOL004954 | isograbrol | 11.04 | 0.5 |
| *licorice* | MOL004955 | isoglabrolide | 14.77 | 0.62 |
| *licorice* | MOL004956 | Isoononin | 8.29 | 0.79 |
| *licorice* | MOL004957 | HMO | 38.37 | 0.21 |
| *licorice* | MOL004958 | Isoschaftoside | 17.38 | 0.83 |
| *licorice* | MOL004959 | 1-Methoxyphaseollidin | 69.98 | 0.64 |
| *licorice* | MOL004960 | 22β-acetylglabric acid | 17.76 | 0.64 |
| *licorice* | MOL004961 | Quercetin der. | 46.45 | 0.33 |
| *licorice* | MOL004962 | 24-Hydroxy-11-deoxyglycyrrhetic acid | 17.57 | 0.76 |
| *licorice* | MOL004963 | 24-Hydroxyglycyrrhetic acid | 24.17 | 0.72 |
| *licorice* | MOL004964 | (Z)-1-(2,4-dihydroxyphenyl)-3-phenylprop-2-en-1-one | 73.18 | 0.12 |
| *licorice* | MOL004965 | 3'(γ,γ-dimethylallyl)-kievitone | 1.21 | 0.63 |
| *licorice* | MOL004966 | 3'-Hydroxy-4'-O-Methylglabridin | 43.71 | 0.57 |
| *licorice* | MOL004967 | 3,3-Dimethylpentane | 41.97 | 0.01 |
| *licorice* | MOL004968 | 3,4,3',4'-Tetrahydroxy-2-methoxychalcone | 1.33 | 0.2 |
| *licorice* | MOL004969 | 2-Ethyl-p-xylene | 20.6 | 0.02 |
| *licorice* | MOL000497 | licochalcone a | 40.79 | 0.29 |
| *licorice* | MOL004970 | 3-methylheptane | 36.61 | 0.01 |
| *licorice* | MOL004971 | 3-methylhexane | 38.19 | 0.01 |
| *licorice* | MOL004972 | 3-Methylpentane | 35.77 | 0 |
| *licorice* | MOL004973 | 3-Ethylpentane | 35.74 | 0.01 |
| *licorice* | MOL004974 | 3'-Methoxyglabridin | 46.16 | 0.57 |
| *licorice* | MOL004975 | 3β-formylglabrolide | 16.36 | 0.55 |
| *licorice* | MOL004976 | Daidzein dimethyl ether | 24.29 | 0.24 |
| *licorice* | MOL004977 | 1-Methoxyficifolinol | 14.61 | 0.86 |
| *licorice* | MOL004978 | 2-[(3R)-8,8-dimethyl-3,4-dihydro-2H-pyrano[6,5-f]chromen-3-yl]-5-methoxyphenol | 36.21 | 0.52 |
| *licorice* | MOL004979 | 4,2',4',alpha-Tetrahydroxydihydrochalcone | 2.45 | 0.16 |
| *licorice* | MOL004980 | Inflacoumarin A | 39.71 | 0.33 |
| *licorice* | MOL004981 | 1-(5-hydroxy-2,2-dimethylchromen-6-yl)-3-(4-hydroxyphenyl)prop-2-en-1-one | 5.2 | 0.34 |
| *licorice* | MOL004982 | 2,6,10-trimethyl-dodecane | 37.8 | 0.03 |
| *licorice* | MOL004983 | 5,6,7,8-Tetrahydro-4-methylquinoline | 59.18 | 0.04 |
| *licorice* | MOL005015 | Licoriisoflavan A | 3.68 | 0.66 |
| *licorice* | MOL004985 | icos-5-enoic acid | 30.7 | 0.2 |
| *licorice* | MOL004986 | 6″-O-acetylliquiritin | 6.26 | 0.82 |
| *licorice* | MOL004987 | 11-deoxyglycyrrhetic acid | 16.21 | 0.76 |
| *licorice* | MOL004988 | Kanzonol F | 32.47 | 0.89 |
| *licorice* | MOL004989 | 6-prenylated eriodictyol | 39.22 | 0.41 |
| *licorice* | MOL004990 | 7,2',4'-trihydroxy－5-methoxy-3－arylcoumarin | 83.71 | 0.27 |
| *licorice* | MOL004991 | 7-Acetoxy-2-methylisoflavone | 38.92 | 0.26 |
| *licorice* | MOL004992 | 7-hydroxy-2-methyl-3-phenyl-chromone | 25.8 | 0.18 |
| *licorice* | MOL004993 | 8-prenylated eriodictyol | 53.79 | 0.4 |
| *licorice* | MOL004994 | 12-methyltetradecanoate | 17.36 | 0.09 |
| *licorice* | MOL004995 | Kanzonol H | 16.92 | 0.8 |
| *licorice* | MOL004996 | gadelaidic acid | 30.7 | 0.2 |
| *licorice* | MOL004997 | Araboglycyrrhizin | 17.73 | 0.14 |
| *licorice* | MOL004998 | Araboglycyrrhizin_qt | 17.71 | 0.74 |
| *licorice* | MOL004999 | Artonin E | 11.38 | 0.8 |
| *licorice* | MOL000500 | Vestitol | 74.66 | 0.21 |
| *licorice* | MOL005000 | Gancaonin G | 60.44 | 0.39 |
| *licorice* | MOL005001 | Gancaonin H | 50.1 | 0.78 |
| *licorice* | MOL005002 | beta-Glycyrrhetinic acid | 17.41 | 0.74 |
| *licorice* | MOL005003 | Licoagrocarpin | 58.81 | 0.58 |
| *licorice* | MOL005004 | Gancaonin I | 21.9 | 0.39 |
| *licorice* | MOL005005 | Glyasperin A | 2.46 | 0.63 |
| *licorice* | MOL005006 | Glyasperins K | 10.15 | 0.44 |
| *licorice* | MOL005007 | Glyasperins M | 72.67 | 0.59 |
| *licorice* | MOL005008 | Glycyrrhiza flavonol A | 41.28 | 0.6 |
| *licorice* | MOL005009 | Corylifolinin | 1.04 | 0.27 |
| *licorice* | MOL005010 | Kanzonol E | 5.77 | 0.71 |
| *licorice* | MOL005011 | Kanzonol Z | 21.77 | 0.76 |
| *licorice* | MOL005012 | Licoagroisoflavone | 57.28 | 0.49 |
| *licorice* | MOL005013 | 18α-hydroxyglycyrrhetic acid | 41.16 | 0.71 |
| *licorice* | MOL005014 | Licorice glycoside A | 5.95 | 0.35 |
| *licorice* | MOL005016 | Odoratin | 49.95 | 0.3 |
| *licorice* | MOL005017 | Phaseol | 78.77 | 0.58 |
| *licorice* | MOL005018 | Xambioona | 54.85 | 0.87 |
| *licorice* | MOL005019 | (2R)-7-hydroxy-2-[4-hydroxy-3-(3-methylbut-2-enyl)phenyl]chroman-4-one | 5.99 | 0.33 |
| *licorice* | MOL005020 | dehydroglyasperins C | 53.82 | 0.37 |
| *licorice* | MOL005021 | Mipax | 57.4 | 0.06 |
| *licorice* | MOL000511 | ursolic acid | 16.77 | 0.75 |
| *licorice* | MOL000561 | Astragalin | 14.03 | 0.74 |
| *licorice* | MOL000057 | DIBP | 49.63 | 0.13 |
| *licorice* | MOL000668 | PENTYLFURAN | 54.59 | 0.02 |
| *licorice* | MOL000671 | ()-Menthol | 59.33 | 0.03 |
| *licorice* | MOL000676 | DBP | 64.54 | 0.13 |
| *licorice* | MOL000703 | 2-heptanone | 46.56 | 0.01 |
| *licorice* | MOL000705 | WLN: VH6 | 19.59 | 0.01 |
| *licorice* | MOL000098 | quercetin | 46.43 | 0.28 |
