# Supplementary material for: Gegen Qinlian decoction enhances the effect of PD-1 blockade in colorectal cancer with microsatellite stability by remodelling the gut microbiota and the tumour microenvironment
Source: Cell Death Dis. 2019 May 28;10(6):415. doi: 10.1038/s41419-019-1638-6 (PMC6538740; doi:10.1038/s41419-019-1638-6)
Supplement: Supplementary file 13 — Supplementary figure legends [file 41419_2019_1638_MOESM13_ESM.docx]

**Supplementary materials**

**Supplementary figure legends for figures S1- S8**

Supplementary Figure S1: Chromatograms of mixed standard solution (A) and sample solution (B). 1: puerarin; 2: daidzin; 3: liquiritin; 4: baicalin; 5: berberine; and 6: wogonoside.

Supplementary Figure S2: PPI network of GQD putative targets.

Supplementary Figure S3: PPI network of known colon cancer-related targets.

Supplementary Figure S4: The interactive PPI network of GQD putative targets and known colon cancer-related targets.

Supplementary Figure S5: Alpha diversity of the faecal microbiome before anti-mouse PD-1 immunotherapy.

Supplementary Figure S6: (A, B, C) Bar plot of compositional differences at the genus level in the gut microbiome of mice from each group. (D, E, F) LDA scores computed for differentially abundant taxa in the faecal microbiomes of mice from each group.

Supplementary Figure S7: Taxonomic cladogram from LEfSe showing differences in faecal taxa. Dot size is proportional to the abundance of the taxon.

Supplementary Figure S8: Unsupervised heatmaps of metabolites significantly changed in the GQD vs. control, PD-1 vs. control and GQD+PD-1 vs. control groups.

**Supplementary tables**

Supplementary Table S1: Monitoring of precursor-to-product ion pairs, declustering potential (DP) and collision energy (CE) of analytes.

Supplementary Table S2: Known colon cancer-related targets.

Supplementary Table S3: Linear regression data and content of analytes.

Supplementary Table S4: Compositive compounds of each compound in GQD.
